# MStoCIRC: A powerful tool for downstream analysis of MS/MS data to predict translatable circRNAs

**DOI:** 10.3389/fmolb.2022.791797

**Published:** 2022-08-22

**Authors:** Zhou Cao, Guanglin Li

**Affiliations:** Key Laboratory of Ministry of Education for Medicinal Plant Resource and Natural Pharmaceutical Chemistry, National Engineering Laboratory for Resource Development of Endangered Crude Drugs in Northwest China, College of Life Sciences, Shaanxi Normal University, Xi’an, China

**Keywords:** circRNA, translation capability, mass spectrometry, MStoCIRC, multifunctional

## Abstract

CircRNAs are formed by a non-canonical splicing method and appear circular in nature. CircRNAs are widely distributed in organisms and have the features of time- and tissue-specific expressions. CircRNAs have attracted increasing interest from scientists because of their non-negligible effects on the growth and development of organisms. The translation capability of circRNAs is a novel and valuable direction in the functional research of circRNAs. To explore the translation potential of circRNAs, some progress has been made in both experimental identification and computational prediction. For computational prediction, both CircCode and CircPro are ribosome profiling-based software applications for predicting translatable circRNAs, and the online databases riboCIRC and TransCirc analyze as many pieces of evidence as possible and list the predicted translatable circRNAs of high confidence. Simultaneously, mass spectrometry in proteomics is often recognized as an efficient method to support the identification of protein and peptide sequences from diverse complex templates. However, few applications fully utilize mass spectrometry to predict translatable circRNAs. Therefore, this research aims to build up a scientific analysis pipeline with two salient features: 1) it starts with the data analysis of raw tandem mass spectrometry data; and 2) it also incorporates other translation evidence such as IRES. The pipeline has been packaged into an analysis tool called mass spectrometry to translatable circRNAs (MStoCIRC). MStoCIRC is mainly implemented by Python3 language programming and could be downloaded from GitHub (https://github.com/QUMU00/mstocirc-master). The tool contains a main program and several small, independent function modules, making it more multifunctional. MStoCIRC can process data efficiently and has obtained hundreds of translatable circRNAs in humans and *Arabidopsis thaliana*.

## 1 Introduction

Circular RNAs (circRNAs) are a covalently closed loop formed by a non-canonical splicing method known as back-splicing. CircRNAs were viewed as by-products of mis-splicing (Cocquerelle et al., 1993), until a circular transcript of the *Sry* gene in mouse testis was identified as a result of normal back-splicing. With the development of next-generation sequencing (NGS) technologies and the establishment of novel algorithms to identify circRNAs on a genome-wide scale (Gao et al., 2015; Ma et al., 2021), circRNAs have been found to be widespread and expressed in specific time and tissue patterns in different organisms (Salzman et al., 2013; Westholm et al., 2014). Flanking intron reverse complementary sequences and ALU repeat sequences contribute to the formation of circRNAs (Jeck et al., 2013; Ivanov et al., 2015). In addition, the types of circRNAs are very rich; they can be classified into exonic circRNAs, intronic circRNAs, intergenic circRNAs, exon-intronic circRNAs, intergenic-exon circRNAs, and intergenic-intron circRNAs (Mehta et al., 2020). Among these circRNAs, exonic circRNAs comprise one or more exons derived from their parental genes and account for the largest proportion (Li S. et al., 2021).

CircRNAs play a fundamental role in the growth and development of organisms. In animals and humans, circRNAs can act as microRNA sponges because the former has one or more binding sites to the latter, preventing microRNAs from interacting with mRNAs in the 3'UTR region (Hansen et al., 2013). CircRNAs can also regulate the expression level of parental genes in some cases by affecting RNA polymerase (Li et al., 2015). In addition, circRNAs have been considered biomarkers in disease prevention (Meng S. et al., 2017; Kristensen et al., 2018). In plants, except for the important roles in plant growth and development, circRNAs can also respond to help plants cope with adversity (Gao et al., 2019; Zhang et al., 2019). As it is speculated, small amounts of circRNAs may be translatable (Abe et al., 2015; Wesselhoeft et al., 2018).

It is reported that researchers have made significant breakthroughs in translatable circRNAs. Since circZNF609 was confirmed to be translatable in 2017 (Legnini et al., 2017), human translatable circRNAs have been revealed one after another (Xia et al., 2019; Jiang et al., 2021). Although the translation ability of ZNF609 is currently controversial (Ho-Xuan et al., 2020), other reported translatable circRNAs convince us that it is worth studying further. circFBXW7 is formed by back-splicing of exons 3 and 4 of the human *FBXW7* gene (Yang et al., 2018) and is found to be translatable. Driven by its upstream internal ribosome entry site (IRES) element (Godet et al., 2019), circFBXW7 is translated into a 185aa peptide (circFBXW7–185aa), which can suppress glioma tumorigenesis. CircEGFR is generated from exons 14 and 15 of the human *ECFR* gene and is shown to translate a novel protein that functions to promote glioblastoma tumorigenesis (Liu et al., 2021). Although the translations of circFBXW7 and circEGFR are both driven by IRES elements, circEGFR is more special than circFBXW7 because circEGFR contains an infinite circRNA open reading framework (icORF) without a stop codon. IcORF on circEGFR enables rolling translation and generates protein products with repeated peptide sequences. In summary, translatable circRNAs really exist in nature, and circRNA-derived peptides have biological functions of great significance.

Furthermore, over the years, biological experiments and computational predictions have been combined to explore scientific questions about translatable circRNAs. In terms of computational prediction, there are some new applications and online databases that can directly provide services for researchers in this field. First, the software applications CircCode and CircPro designed their unique algorithms to fully utilize ribosome profiling (ribo-seq) to predict translatable circRNAs (Mumtaz and Couso, 2015; Meng X. et al., 2017; Sun and Li, 2019). These tools have predicted hundreds of translatable circRNAs from different species. Second, the circRNADb database is an earlier established and comprehensive online database that analyzes information about microRNA (miRNA) binding sites, circRNA open reading framework (cORF) sequences, and IRES locations on circRNAs (Chen et al., 2016). The latest online databases riboCIRC and TransCirc are more powerful and specialized because they have collected and analyzed as much evidence as possible (Li H. et al., 2021; Huang et al., 2021), including both direct and indirect pieces of evidence, such as ribo-seq, mass spectrometry (MS), IRES elements, N6-methyladenosine (m6A) modification (Meyer et al., 2015; Yang et al., 2017), and translation initiation site (TIS) (Hernández et al., 2019). After a comprehensive assessment of the translation potential of circRNAs, tens of thousands of translatable circRNAs of higher confidence have been proposed in their research results, which make a great contribution to this field.

Unfortunately, despite the increasing abundance of mass spectrometry data, there is still a lack of tools to utilize them rationally. In PRIDE, raw mass spectrometry data cover different species, different periods of the same species, and even different strategies for the same experimental material (Shao and Lam, 2017; Ankney et al., 2018). This inspires us to build up a novel translation circRNA prediction pipeline based on raw mass spectrometry data. Mass spectrometry data can serve as strong evidence to support the translation of different complex templates. Before our work, several studies had focused on the utility of mass spectrometry data by combining proteomics and other omics to identify novel genes (Nakayama et al., 2011), even translatable non-coding RNAs (Giambruno et al., 2018). In other words, the prediction of translatable circRNAs based on mass spectrometry evidence is feasible. Therefore, we designed a scientifically efficient pipeline and implemented an equivalent analysis tool named mass spectrometry to translatable circRNAs (MStoCIRC).

MStoCIRC is an analysis tool based on mass spectrometry to predict the translation capacity of circRNAs. Its input files come from mass spectrometry analysis software applications, such as pFind, MaxQuant, and Mascot (Li et al., 2005; Cox and Mann, 2008), and are considered the backbone, while other pieces of evidence are considered in the following steps. As MStoCIRC gradually combines multiple pieces of evidence, the number of translatable circRNAs decreases, and the ones that survive in the end represent the most likely translatable circRNAs. In addition, despite not being relevant to direct evidence, some extra function modules are important and have also been implemented, such as merging mass spectrometry-based (MS-based) peptides, into the longest peptides. The longer the peptide spanning the back-splice junction sites (BSJs), the more possibly circRNAs are translatable in nature. Compared with current applications and databases, MStoCIRC has several advantages: 1) since it is an offline tool connecting mass spectrometry with circRNAs, users can flexibly operate MStoCIRC according to their research interests to predict translatable circRNAs; and 2) we designed some extra functions in MStoCIRC to make the predicted results more reliable.

## 2 Materials and methods

**FIGURE 1:**
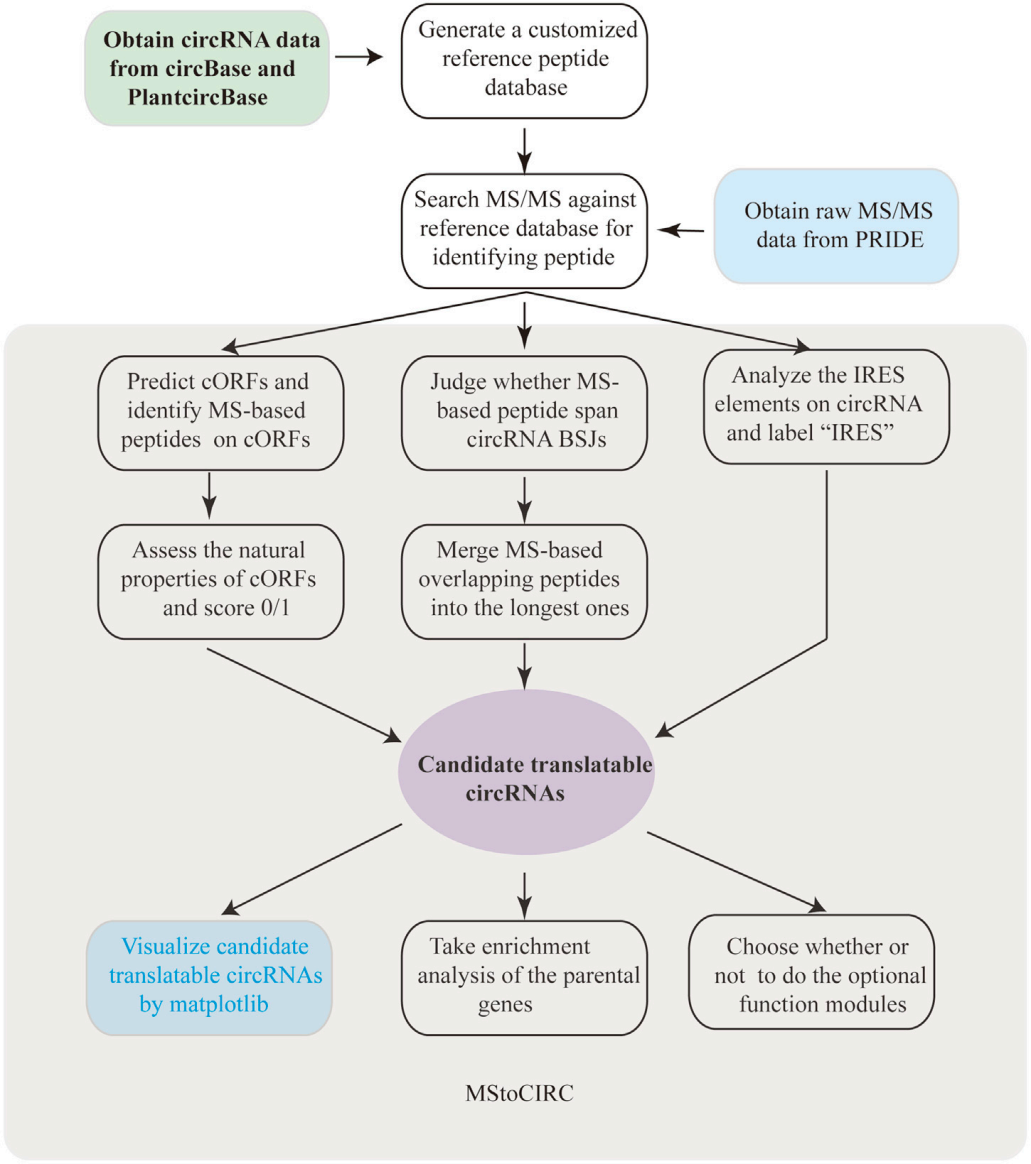
Workflow of MStoCIRC.

The analysis pipeline of MStoCIRC used to identify translatable circRNAs has been established and shown in Figure 1. In the beginning, we aimed to implement MStoCIRC only *via* the Python3 programming language, until the R package “clusterProfiler” is found to have advantages over the Python3 module in GO and KEGG analyses (Yu et al., 2012; Anders et al., 2015), which forced us to program by the R language in this function module. In addition, the implementation of MStoCIRC also relies on some important Python3 modules. For example, by installing the “matplotlib” module (Rajaei et al., 2021), MStoCIRC can visualize the predicted results with complex graphs.

### 2.1 Identification of MS-based peptides from raw MS/MS data

**FIGURE 2:**
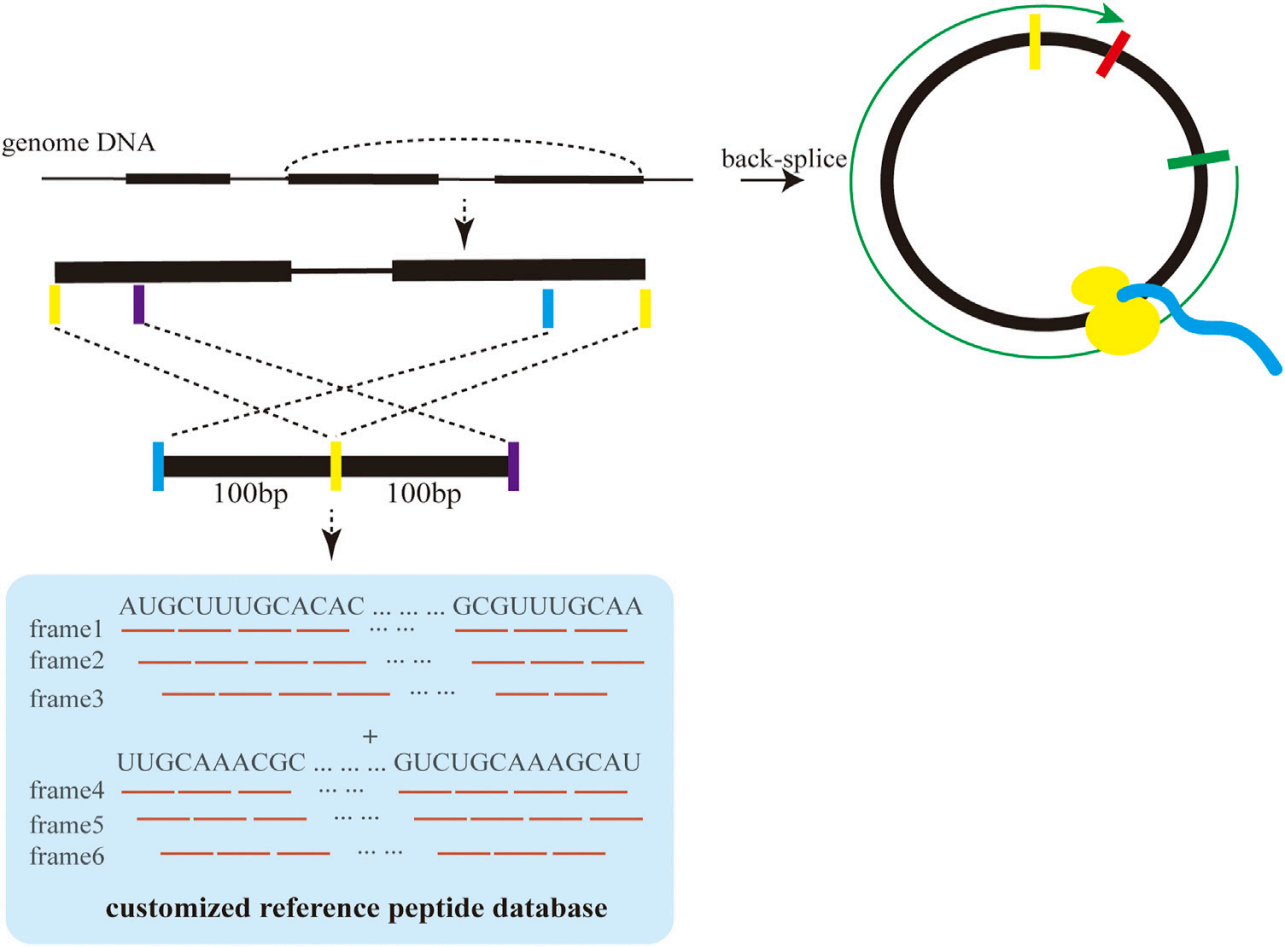
Construction of the reference peptide database based on circRNA sequences.

These raw MS/MS data can be downloaded from online databases, such as PRIDE (https://www.ebi.ac.uk/pride/archive) or obtained from other channels (Perez-Riverol et al., 2019). First, we selected the corresponding mass spectrometry software pFind because of its stable operation and high reliability in processing raw MS/MS data. Second, the reference peptide sequence database was prepared by translating a nucleotide sequence of about 200 bp around the BSJs of circRNAs in six reading frames shown in Figure 2 (Wang et al., 2020). Third, pFind was used to search raw MS/MS data against the customized reference peptide sequence database for the identification of proteins and peptides.

### 2.2 Identification of circRNA-derived peptides supported by MS-based peptides

**FIGURE 3:**
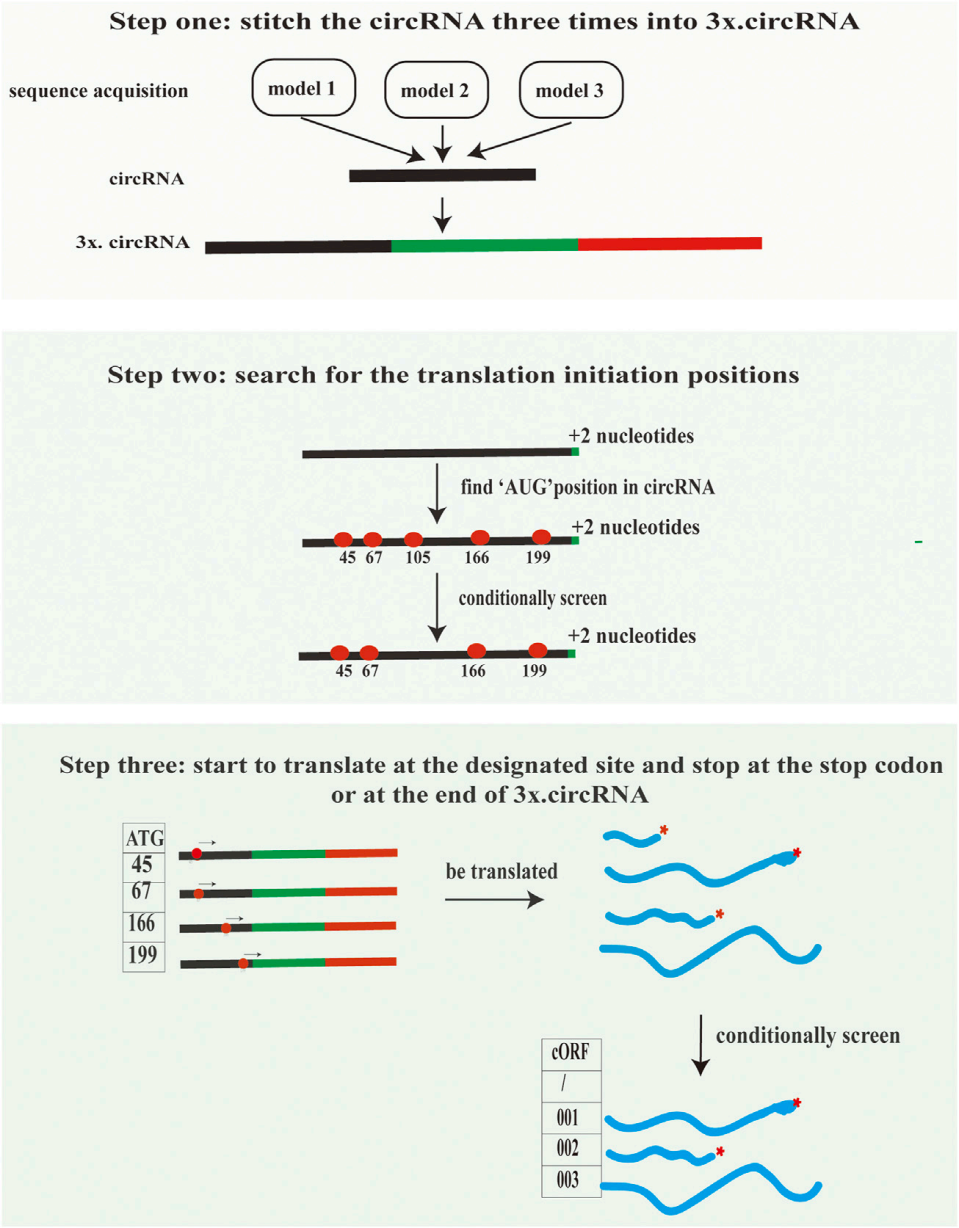
Algorithm used to identify all possible cORFs in each circRNA. Models 1 to 3 show three approaches for obtaining the exon nucleotide sequences of circRNAs to predict cORFs. In step 1, the sequences of circRNA are repeated three times so that the cORFs in circRNAs can be predicted completely, except for those cORFs rolling the circle more than three times. In step 2, “+2 nucleotides” in the 3' end of the sequence can avoid missing start codons located at BSJs (e.g., “A^TG” and “AT^G”) when searching for possible start codons in circRNA sequences. In the final step, all the possible cORFs are predicted.

This module can assist in inferring the translation of circRNAs supported by mass spectrometry evidence. The accomplishment of this module consists of three steps. First, the exon nucleotide sequences of circRNAs are indispensable for MStoCIRC. Also, MStoCIRC has three models to obtain sequences. In model one, it flexibly skips the first step when the exon sequence of circRNAs is entered straightforwardly by setting the parameter “--sequence.” Otherwise, the parameter “--info” must be satisfied. MStoCIRC also has other two different models to extract the exon sequences of circRNAs. Model two is to extract exon sequences of circRNAs based on exon numbers and positions referred to the genome annotation file (e.g., genomic. gtf). Model three relies on transcript files, that is, mature messenger RNA (mRNA) sequence files with intron sequences are excluded. When the start and end sequences of exonic circRNAs are determined, the exon sequences of circRNAs can be extracted from the corresponding mRNA sequences. Here, we recommend the latter approach since for a considerable number of species, the splicing signals are complex (Pan et al., 2008), making it difficult to correctly extract sequences with genome annotation files. Second, all putative cORFs are predicted and translated into putative peptide sequences. The putative peptide sequences with a length of more than 20 amino acids and an initiation codon of “AUG” will be defined as possible circRNA-derived peptides. We designed the algorithm to predict all possible longest cORFs belonging to each circRNA (Figure 3) and implemented it in Python3 without installing additional applications while running “ORF_pipeline_predict.py” depending on bedtools (Pamudurti et al., 2017). We compared the predicted possible circRNA-derived peptides in the NCBI ORFfinder (https://www.ncbi.nlm.nih.gov/orffinder/) to verify the accuracy and completeness of our predicted circRNA-derived peptides. Third, the MS-based peptides were mapped onto circRNA-derived peptides to obtain meaningful MS-based peptides, and those that could not be successfully mapped were discarded.

### 2.3 Assessment of natural properties of circRNA-derived peptides

Not all predicted cORFs are inherently translatable. This is not only because circRNAs have multiple putative cORFs but also because some of their translation products may be easily degraded and undetectable. We attempted to identify cORFs with a higher probability of translation by a machine learning strategy (Greener et al., 2022). As for datasets of machine learning, the positive dataset consists of mRNA-related proteins and the non-coding RNA-encoded peptides from ncEP, and the negative dataset includes the peptides generated randomly. These datasets are then mixed sufficiently and randomly into a whole dataset, and the whole dataset is divided by 8:2 into training datasets and test datasets. Then, both the training datasets and test datasets were transformed into the standard form. The training dataset is used to train the Naive Bayes model of Scikit-learn, and the test dataset is used to evaluate the reliability of the selected model and calculate the accuracy. Finally, all cORFs of circRNAs were analyzed and given a score of 0/1.

### 2.4 Judgment on whether the MS-based peptides span the back-splice junction sites

CircRNAs are generated by a special back-splicing method. Non-coding RNAs (ncRNAs) include miRNAs, long non-coding RNAs (lincRNAs), and rRNAs (Mohr and Mott, 2015; Kopp and Mendell, 2018), and the special back-splicing method enables circRNAs to be distinguished from these ncRNAs. Therefore, the nucleotide sequences around BSJs are frequently applied to identify circRNAs in transcriptome sequencing (RNA-seq), and in this function module, we identified translatable circRNAs by judging whether the MS-based peptides span BSJs or not. As mentioned previously, the reference peptide sequence database was made by translating the nucleotide sequence around BSJs into six frames, and the BSJs are expected to appear in the middle of the reference peptide sequences. The MS-based peptide was mapped to the reference peptide sequence, in order to determine whether the MS peptides spanned the BSJs. Well-matched peptides were retained for further analysis, and the term “YY”+num was created to indicate at least how many amino acids of MS-based peptides were retained on either side of the BSJs. For example, “YY4” means that at least four amino acids were found before and after BSJs. In general, the larger the value, the more likely the circRNAs are to be translated.

### 2.5 Merging of the overlapping MS-based peptide into the longest one

The translation possibility of circRNAs can be reflected by the coverage of circRNA-derived peptides in mass spectrometry analysis, the number of different MS-based peptides which span the same BSJs, and the length of the merged MS-based peptide. To perform this, we extended the overlapping MS-based peptides together. We implemented this process through an efficient strategy. After the MS-based peptides were sorted by merging, they were sorted into different two-dimensional lists according to circRNA names and their cORF numbers. Peptides belonging to the same one-dimensional list are then sequentially mapped onto the same cORF in a circular manner until all of these peptides have been used over. For each cycle, the start and end indexes of mapping MS-based peptides onto cORFs were recorded and compared with the index values of the previous cycle. The start index is replaced by the smaller value, and the end index is substituted by the larger value. The final start and end indexes were able to reflect the longest merged MS-based spanning BSJ peptides.

### 2.6 Prediction of the internal ribosome entry site elements on circRNAs

FASTA files are taken as input files and used to determine whether IRES elements are present on circRNA sequences. Due to the poor conservation of IRES elements, complex spatial structures, the features of uncertain sequence lengths, and unmentioned other features, finding unknown IRES elements is a huge challenge. Therefore, instead of *de novo* programming implementation, we evaluated the IRES element on circRNAs by using published applications (e.g., VIP, IRESPred, IRESfinder, and IRESpy) (Hong et al., 2013; Kolekar et al., 2016; Zhao et al., 2018; Wang and Gribskov, 2019). We decided to integrate the application IRESfinder (Zhao et al., 2018) into the “IRES_predict” function module. IRESfinder is widely recognized to predict IRES elements on eukaryotic genomes. Programmed by the Python3 language, it can accurately and efficiently predict elements through machine learning by using experimentally validated IRES sequences as a positive subset. Then, circRNAs with an IRES prediction score >0.5 were written to a summary file labeled “IRES” and vice versa. circRNAs labeled “Non-IRES” were also retained because these circRNAs may also have translation potential and may rely on other cap-independent translation mechanisms, such as m6A-mediated translation.

### 2.7 Enrichment analysis for parental genes

Mass spectrometry analysis makes it possible to identify hundreds of novel circRNAs that encode peptide products with fundamental biological functions. Also, these translatable circRNAs may originate from different tissues and growth phases. Here, we performed GO term and KEGG pathway analyses on the parental genes of predicted translatable circRNAs. In this way, the relationship between the parental genes of circRNAs and the metabolic pathways circRNA-derived peptides may participate in will be clearly described (Yang et al., 2018; Jiang et al., 2021; Liu et al., 2021). Furthermore, Sgroi et al. (2020) described an important pathway in which well-known cancer genes (*MAPK1*/*ATK3*/*EGFR*) were covered. In this pathway, at least three genes were parental genes of experimentally validated and published translatable circRNAs, which inspired us to perform GO and KEGG analyses. MStoCIRC can also perform enrichment analysis and save results in pictures for researchers (Supplementary Material). This function module is programmed by the R language rather than the Python3 language because the R package “clusterProfiler” is well designed.

### 2.8 Visualization of the predicted results

The “matplotlib” Python3 module is needed in this step because “matplotlib” specializes in drawing complex and beautiful pictures for research. The visualization scheme of translatable circRNAs is designed not only to highlight the structure of translatable circRNAs but also to facilitate user understanding. For example, for some translatable circRNAs whose cORF rolls circle more than one time, the half diameter gradually increases to draw cORF circles that look like a spiral. The visualization parameters are collected gradually *via* the aforementioned steps and written into “circ_draw.txt”. For example, IRES elemental analysis adds parameters of the IRES location on circRNAs. Two pictures of SVG format for each translatable circRNA are saved in the “draw_circ” folder.

### 2.9 Other optional functions

Some optional function modules are also provided, and users can choose these modules according to their actual needs, making MStoCIRC more powerful and personalized. The “Rem_peptide” module is to remove repeated MS-based peptide sequences so that the remaining sequences differ from each other. The “Map_gene” module can map MS-based peptides to linear proteins of parental genes to prevent the case that peptides spanning BSJs actually originating from linear proteins because of the degeneracy of codons. Moreover, because current research indicates that circRNA-derived peptides are related to the biological function of the parental gene (Yang et al., 2018; Xia et al., 2019; Liu et al., 2021), the “Circ_annote” module annotates the potential biological function of circRNA-derived peptides according to the parental gene. The “Ms_ribo” module can judge whether the predicted translatable circRNAs have ribosome profiling evidence from the nucleotide sequence directly entered in the FASTA format. The “CircRNA_classify” module classifies the predicted translatable circRNAs into six classes *via* two criteria. One criterion is the number of cORFs rolling the circle, and circRNAs are divided into three groups, the “less than one lap” group, “less than two laps” group, and “more than two laps” group. As for circRNAs of group 3, their cORFs have no stop codons, roll circles from beginning to end, and are always translated into polypeptides containing repeated sequences. Another criterion is the presence of IRES elements corresponding to cORFs in translatable circRNAs. Therefore, the translatable circRNAs of class I mean that the cORF lengths are smaller than the full lengths of circRNAs (cORF length/circRNA full length <1), and the translation process may be driven by IRES elements. The reason why we classified circRNAs into different categories is that it can help researchers to select ideal circRNA research subjects.

## 3 Results and discussion

Research on translatable circRNAs has increased in recent years. The biological functions of published circRNA-derived peptides and the increasing number of circRNAs with unknown biological functions highlight the research significance and demand more effort and energy on translatable circRNAs. Meanwhile, the methods related to computational prediction are basically several tools and online databases, which are more inclined to ribosome profiling statistics. Compared to ribosome profiling, protein mass spectrometry analysis for translatable circRNAs is also an effective strategy, while the protein mass spectrometry data uploaded online are very large and abundant and may not be fully used. In this study, we developed a tool MStoCIRC to identify translatable circRNAs based on raw mass spectrometry data. MStoCIRC not only comprehensively uses as much evidence as possible but also carefully considers the characteristics of raw MS/MS data. As it may be one of the few tools for predicting translatable circRNAs based on mass spectrometry, we cannot make in-depth comparisons with other tools to describe its relatively large advantages but can only emphasize its future potential.

This research aims to implement more function modules in MStoCIRC in order to make the computational prediction of translatable circRNAs more efficient and accurate (Table 1). The function modules have been optimized, including sorting statistics by the Merge Sort algorithm and adding multiple threads. As depicted in “identify the MS-based peptides on circRNA-derived peptides”, there exist three models of MStoCIRC to extract the exonic nucleotide sequences of circRNAs and predict all putative cORFs for each circRNA. Under model 1, it took less than 7 min to analyze more than 30,000 circRNAs based on more than 30,000 MS-based peptide sequences, and the running time has been decreased five-fold. The IRES prediction function module, as an integral part, accounted for the largest proportion of the total time cost.

**TABLE 1:** Functional description of MStoCIRC.

Feature	Function name	Description of the function
Major function	map_corf	Map MS-based peptides onto circular RNA open reading frameworks (cORFs)
	sklearn_coding	Assess the natural properties of cORFs
	map_junct	Judge whether MS-based peptides span BSJs
	peptide_merge	Merge the overlapping MS-based peptides across BSJs into longest ones
	ires_predict	Predict IRES elements on circRNAs
	path_analysis	Perform enrichment analysis
	draw_circ	Visualize the sequence information of translatable circRNAs
Optional function	rem_peptide	Remove repeated MS-based peptides
	map_gene	Map MS-based peptides onto linear proteins
	circ_anno	Annotate biological functions according to parental genes
	ms_ribo	Add pieces of evidence of translation
	circ_classify	Classify translatable circRNAs into six classes

### 3.1 Performance of MStoCIRC

The false discovery rate (FDR) value is a necessary component of performance evaluation for most tools (Sun and Li, 2019), and the FDR of MStoCIRC is also calculated by the target-decoy approach described as follows (Elias and Gygi, 2007). Virtual circRNA sequences are reversely complementary sequences of the circRNA downloaded from circBase (http://circrna.org/) (Glažar et al., 2014). Therefore, these virtual sequences do not actually exist in nature and are considered equivalent to the real circRNA sequences. MStoCIRC then analyzed them as before and on the condition that MS-based peptides span BSJs more than two amino acids, it generated predicted final results containing very few translatable circRNAs (26 translatable circRNAs), whereas about 540 translatable circRNAs were obtained from 140,742 circRNA sequences. The calculated FDR value is less than 0.05, which is a very low value that can strongly convince us that MStoCIRC has a scientific and rigorous analysis pipeline and can predict the results accurately and reliably.

**FIGURE 4:**
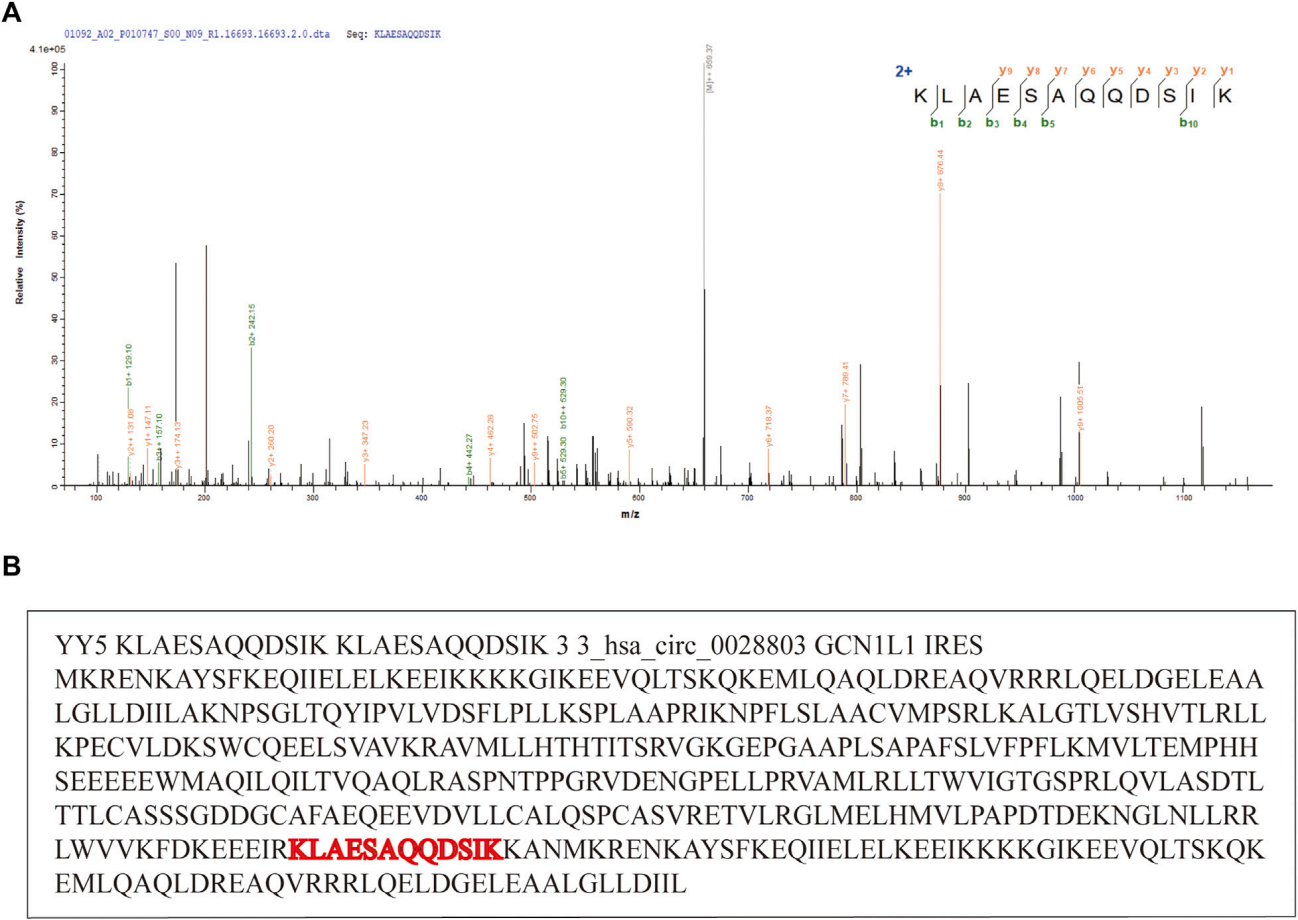
Example of the translatable circRNA, hsa_circ_0028803. (A) Evidence of that circ_028803 is a translatable circRNA. “KLAESAQQDSIK” is the peptide spanning hsa_circ_028803 BSJ. (B) Predicted details about the translatable circRNA hsa_circ_0028803.

In addition to FDR evaluation, we also collected experimentally identified and published circRNAs as a positive set. As we expected, pFind identified numerous MS-based peptide spectra against the reference peptide sequence database. Then, MStoCIRC sensitively found the longest MS-based peptide spanning the BSJs and successfully predicted four published translatable circRNAs. Take hsa_circ_0028803 for example, the MS-based peptide sequence which is a peptide-spectrum-match (PSM) result of the highest score was identified by pFind and visualized by pLabel, and MStoCIRC further analyzed and re-identified the translatable hsa_circ_0028803 from the positive set (Figure 4).

### 3.2 Visualization result of translatable circRNAs

**FIGURE 5:**
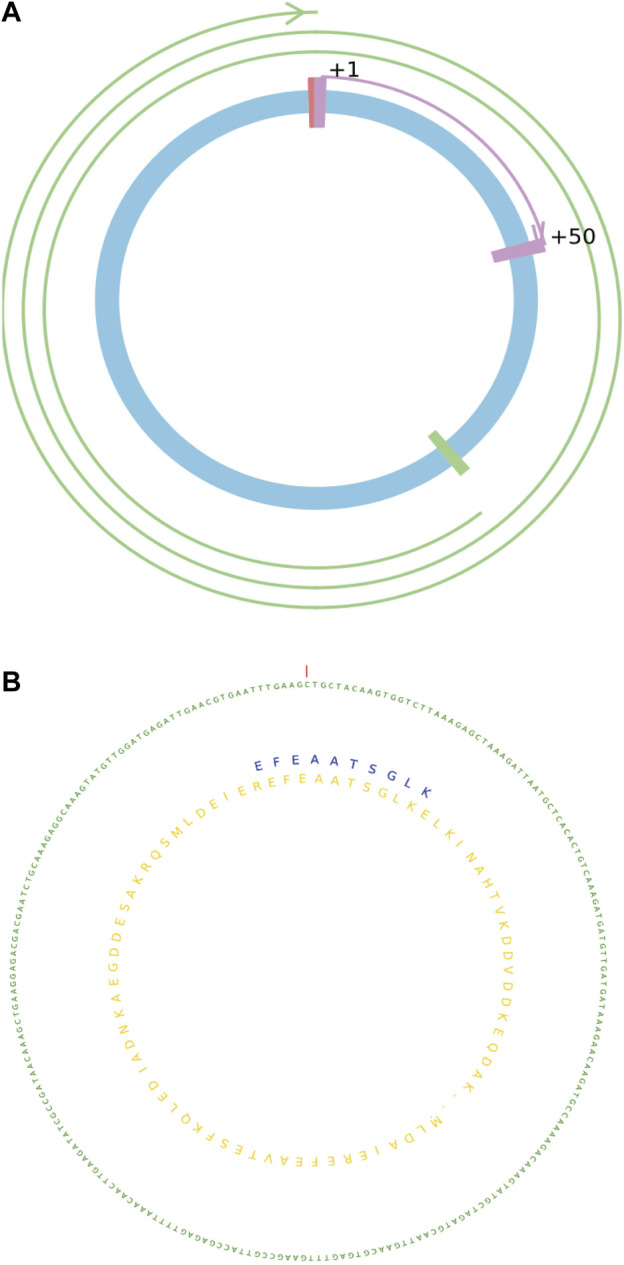
Visualization of the translatable circRNAs with details. **(A)** cORF information on the translatable circRNA. **(B)** Sequence information on the translatable circRNA with the translated peptide or protein.

MStoCIRC can visualize the predicted details of translatable circRNAs from two different angles (Figure 5). One way is to use lines and curves of different colors, lengths, and thicknesses to describe the cORF information on translatable circRNAs. From another angle, nucleotide and amino acid sequences of different colors are shown and labeled. Visualization model can be applied for different translatable circRNAs although the predicted results are complex, such as the numbers of cORFs rolling the circles ranging from <1 to >2, IRES can be located anywhere in the circRNA, and the full lengths of circRNAs can be more than 1,000 bps.

### 3.3 Multifunctional tool for users

To make MStoCIRC more versatile, the modules in MStoCIRC are programmed separately and assigned in different folders. The modules could be invoked by the main program and used in the terminal command line when needed. For example, to predict the cORFs, it would be a better choice of running the “ORF_predict.py ” module rather than running the entire established modules in MStoCIRC.

### 3.4 Predicted translatable circRNAs in humans and *Arabidopsis thaliana*

In order to show the application of MStoCIRC, we considered humans and *Arabidopsis thaliana* as examples to identify translatable circRNAs. Raw MS/MS data and circRNA information on humans and *Arabidopsis thaliana (A. thaliana)* were first downloaded (Chu et al., 2017). After successfully running MStoCIRC for computational prediction, we obtained in total 1,039 translatable circRNAs of humans (Supplementary Material/result. mstocirc.hsa) and 620 translatable circRNAs of *A. thaliana* (Supplementary Material/result. mstocirc.ath). Due to the suitable molecular weight of circRNA-derived peptides, the high score of IRES elements, and the origin of parental genes with significant biological functions, some predicted circRNAs may be excellent subjects for related research studies. These predicted translatable circRNAs can be used as materials for biological experimental validation, which can reduce much workload.

## 4 Conclusion

Mass spectrometry can, like ribosome profiling, serve as a profound method to underpin the translation function of circRNAs. CircCode and CircPro are tools that conduct similar research by focusing on ribosome profiling analysis. However, tools that directly connect protein mass spectrometry to translatable circRNAs are still lacking. MStoCIRC initially processes the preliminary results generated by mass spectrometry, then takes serial scientific analysis referred to in the established analysis pipeline, and finally obtains the comprehensive predicted results of translatable circRNAs. MStoCIRC is a promising tool not only because it is feature-rich and makes the final results more reliable but also because it is an offline tool that provides users with an interface to flexibly predict translatable circRNAs with comprehensive evidence. MStoCIRC as a tool for the downstream analysis of raw MS/MS data makes better use of the raw MS/MS data stored in the online database. To test the performance, humans and *A. thaliana* were used as model species for animals and plants, respectively. Finally, it is not impossible to analyze other species; for this, users only need to change the input file format to one that MStoCIRC can recognize.

## Data Availability

The datasets presented in this study can be found in online repositories. The names of the repository/repositories and accession number(s) can be found in the article/Supplementary Material.
