# Supplementary figures and images for: MStoCIRC: A powerful tool for downstream analysis of MS/MS data to predict translatable circRNAs
Source: Front Mol Biosci. 2022 Aug 22;9:791797. doi: 10.3389/fmolb.2022.791797 (PMC9441560; doi:10.3389/fmolb.2022.791797)

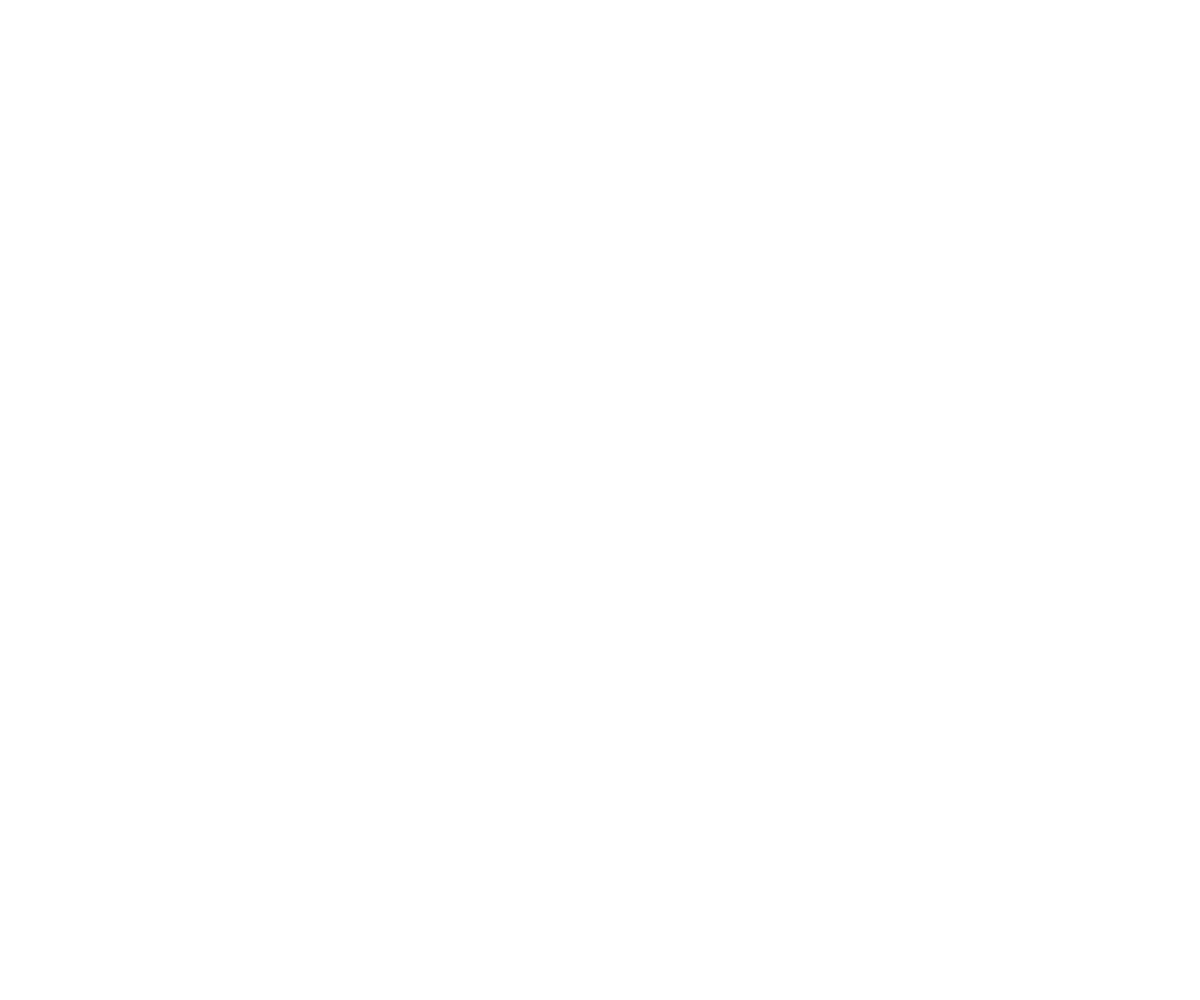

Supplement: Supplementary file 1 [file DataSheet1.ZIP › Supplementary_Materials/result.mstocirc.ath/5enrich/1.jpg]

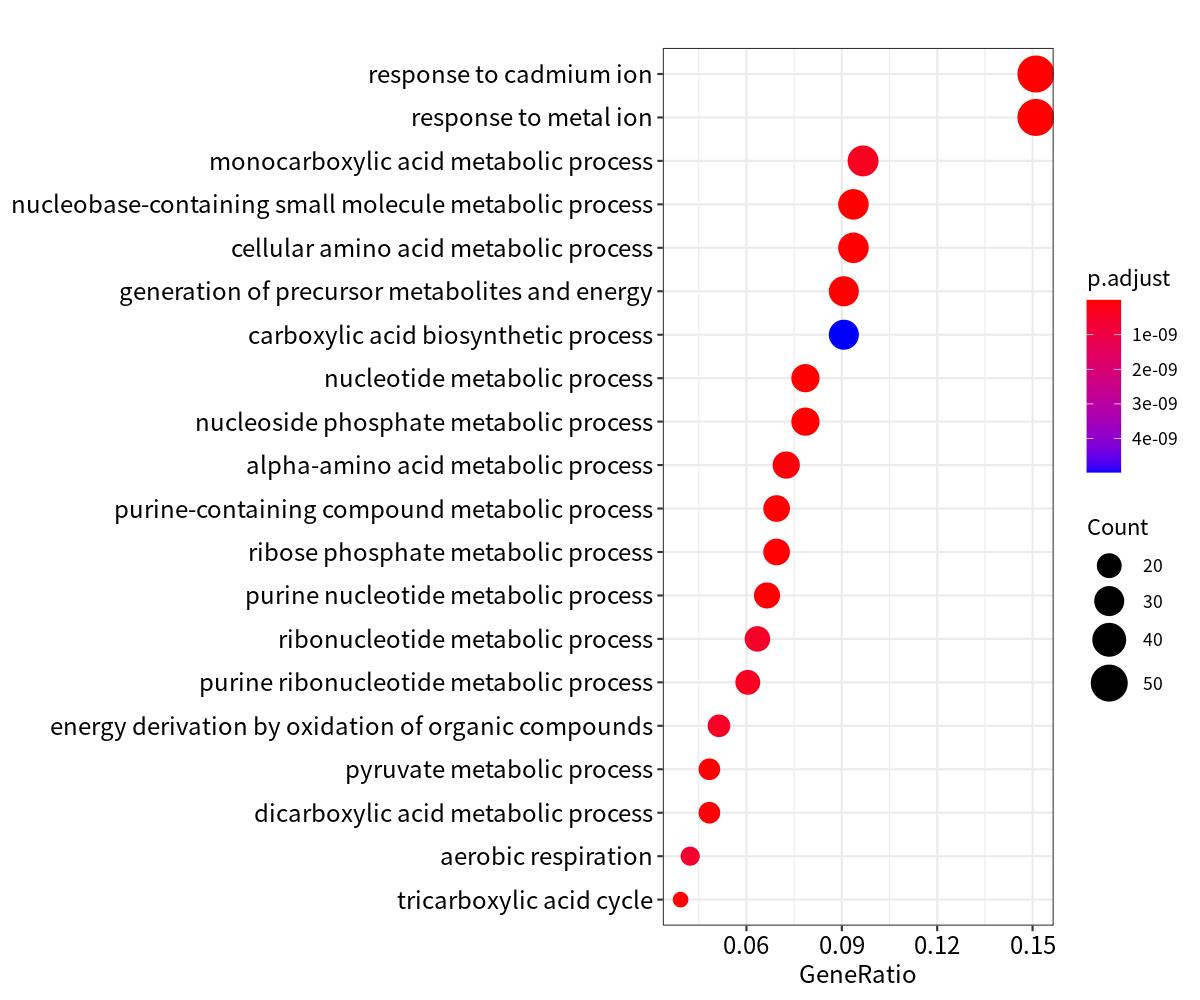

Supplement: Supplementary file 1 [file DataSheet1.ZIP › Supplementary_Materials/result.mstocirc.ath/5enrich/2.jpg]

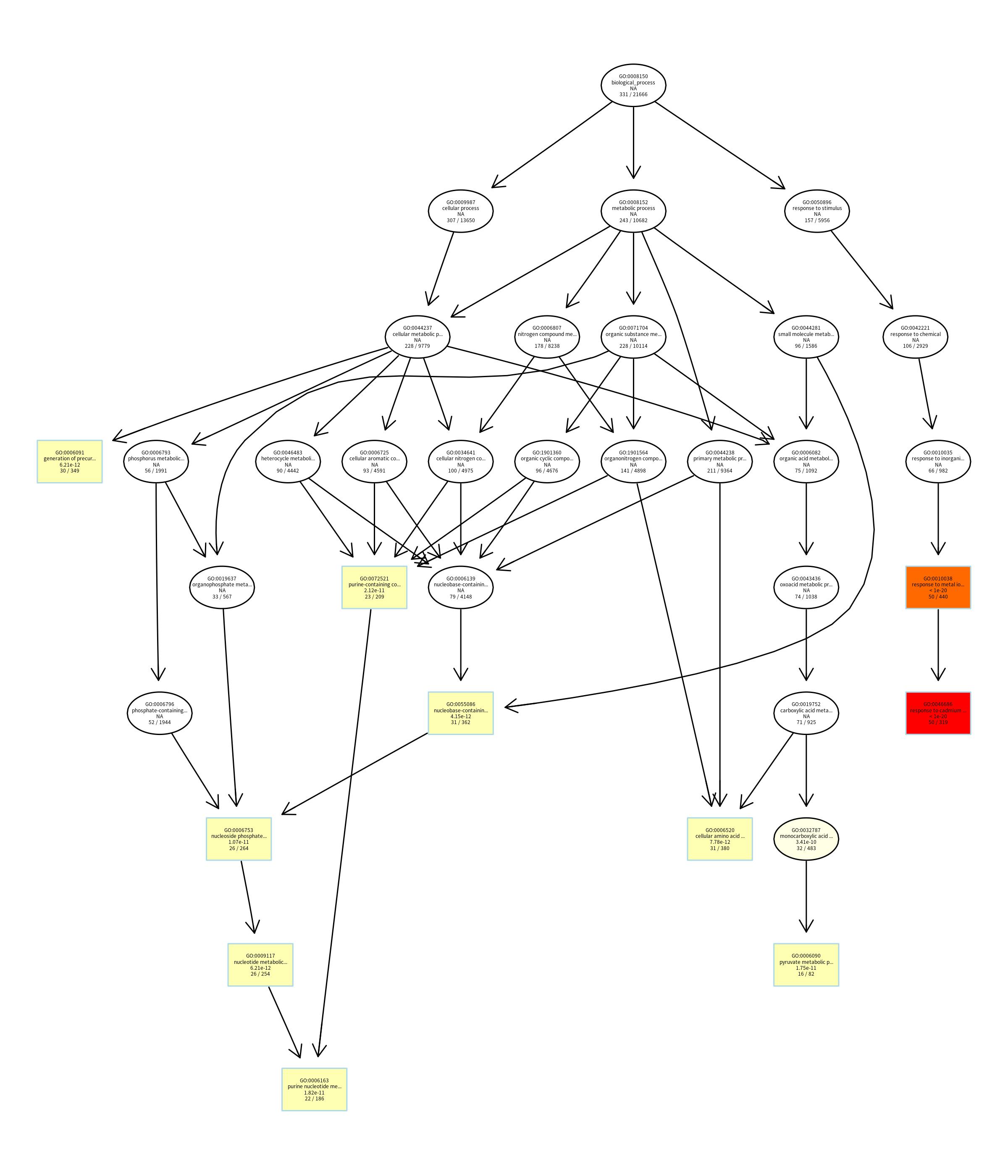

Supplement: Supplementary file 1 [file DataSheet1.ZIP › Supplementary_Materials/result.mstocirc.ath/5enrich/3.jpg]

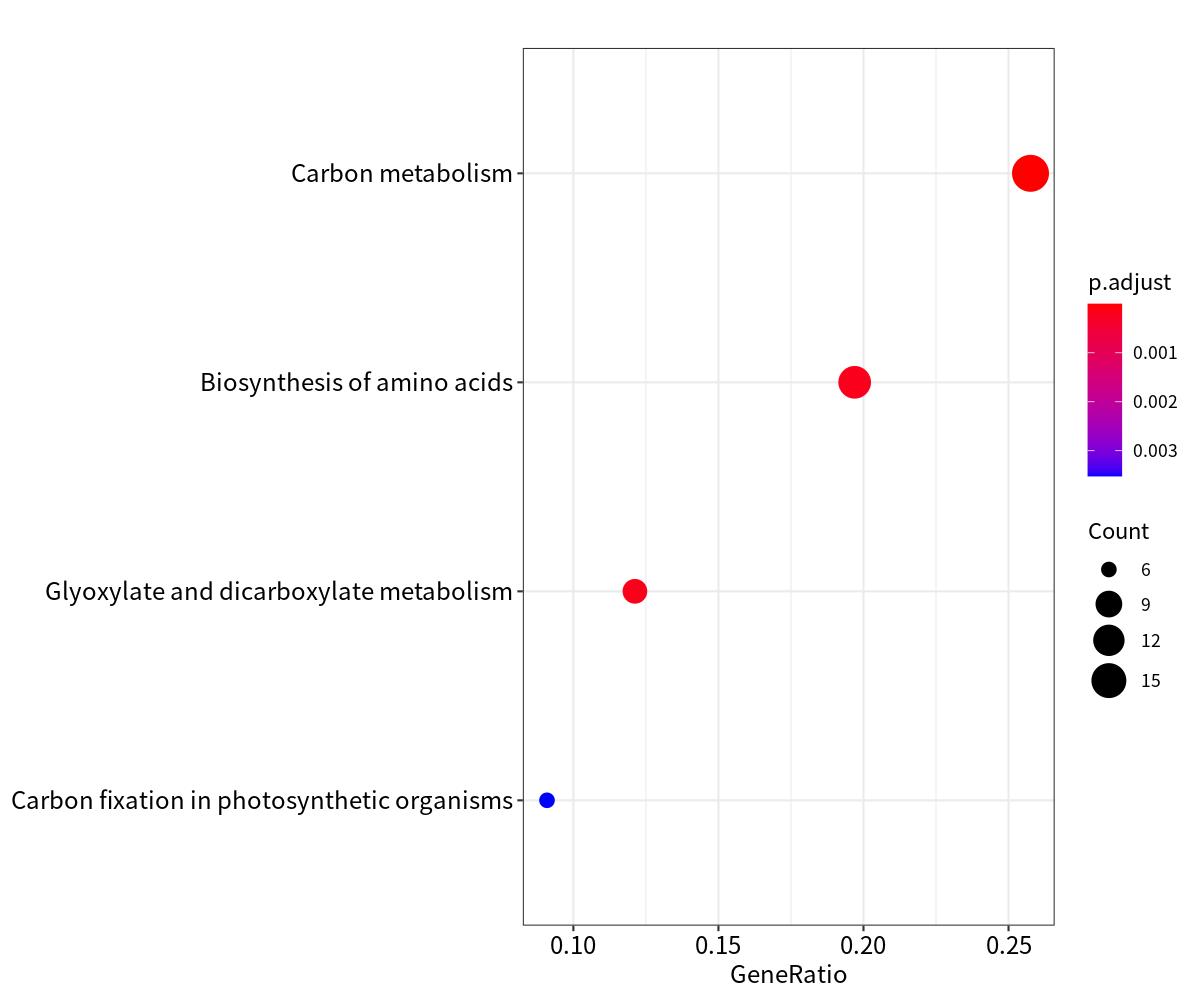

Supplement: Supplementary file 1 [file DataSheet1.ZIP › Supplementary_Materials/result.mstocirc.ath/5enrich/4.jpg]

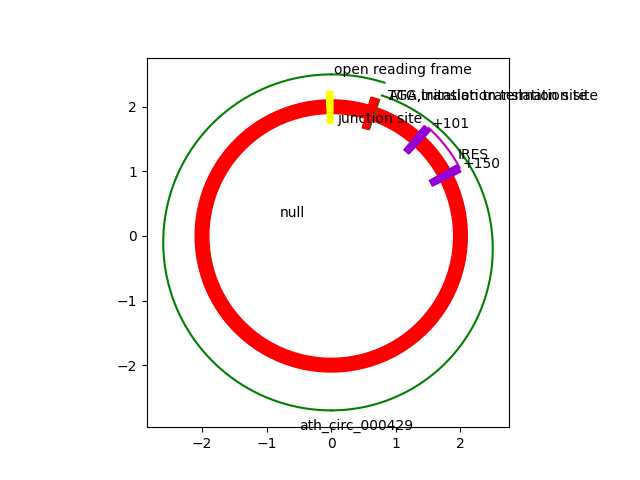

Supplement: Supplementary file 1 [file DataSheet1.ZIP › Supplementary_Materials/result.mstocirc.ath/6draw_circ/ath_circ_000429_circc.png]

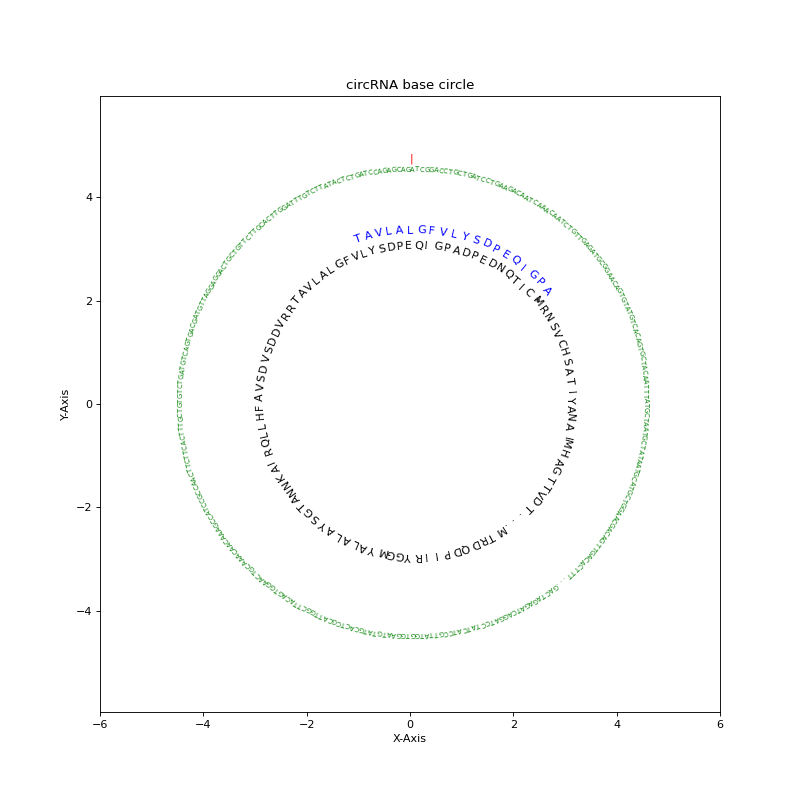

Supplement: Supplementary file 1 [file DataSheet1.ZIP › Supplementary_Materials/result.mstocirc.ath/6draw_circ/ath_circ_000429_circw .png]

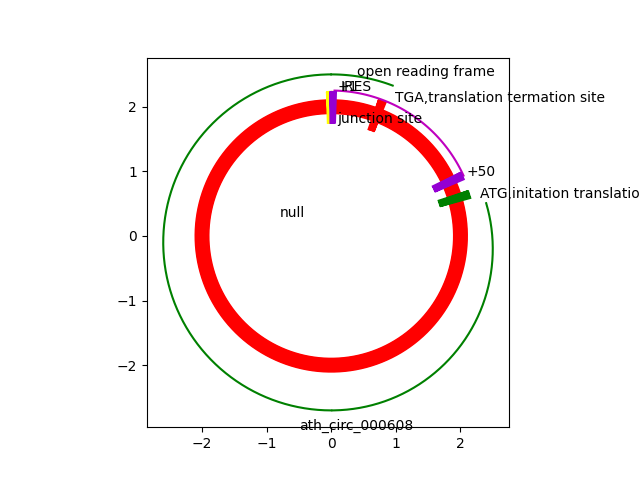

Supplement: Supplementary file 1 [file DataSheet1.ZIP › Supplementary_Materials/result.mstocirc.ath/6draw_circ/ath_circ_000608_circc.png]

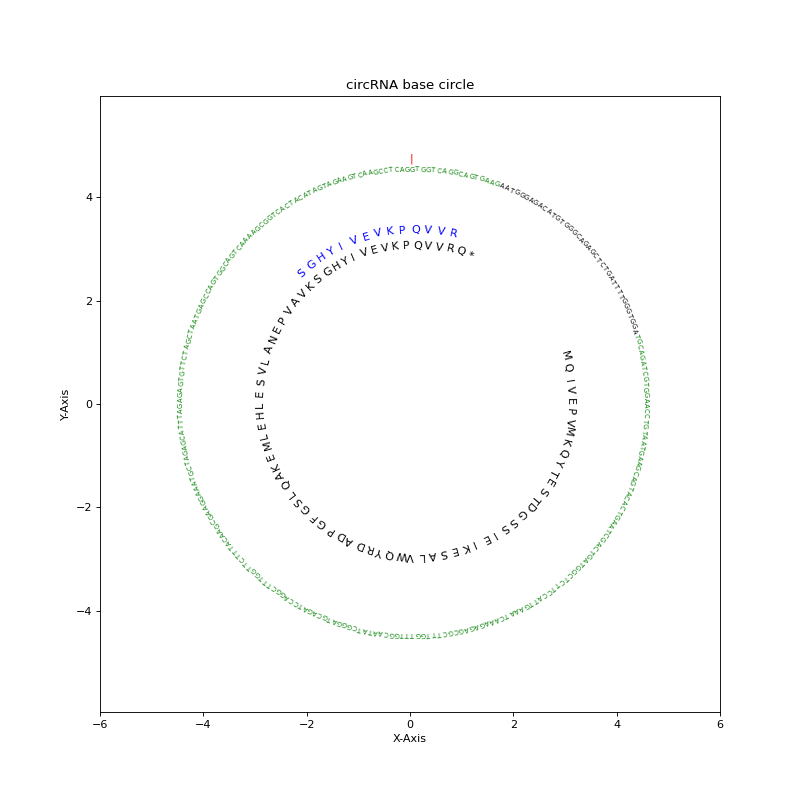

Supplement: Supplementary file 1 [file DataSheet1.ZIP › Supplementary_Materials/result.mstocirc.ath/6draw_circ/ath_circ_000608_circw .png]

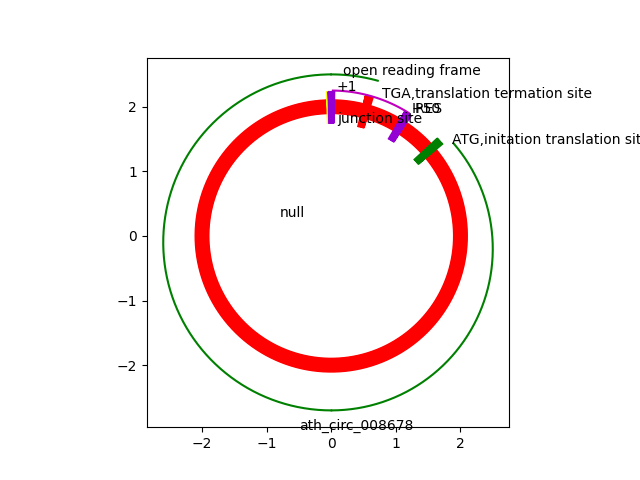

Supplement: Supplementary file 1 [file DataSheet1.ZIP › Supplementary_Materials/result.mstocirc.ath/6draw_circ/ath_circ_008678_circc.png]

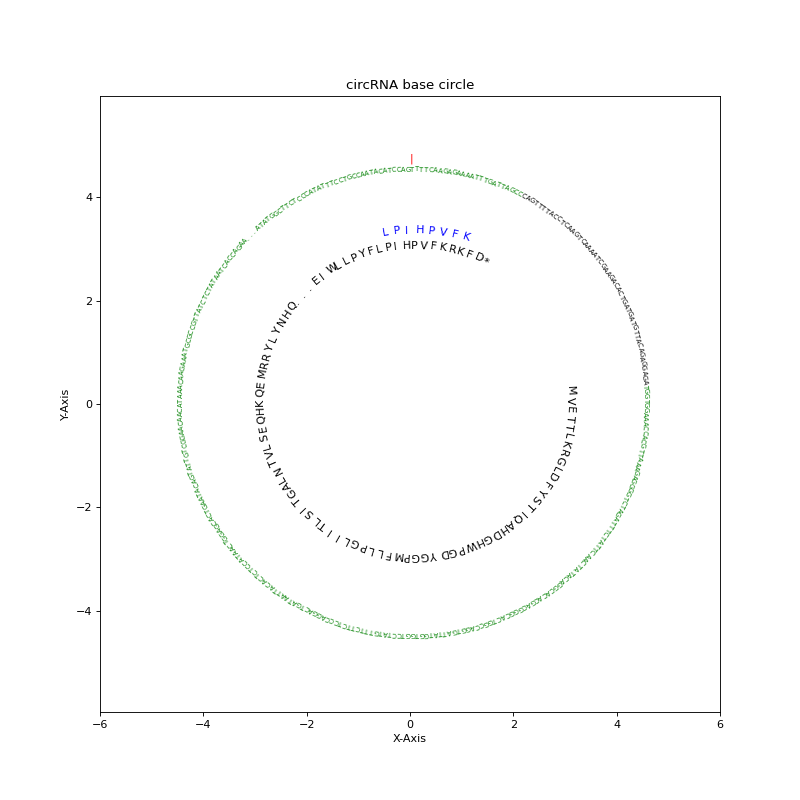

Supplement: Supplementary file 1 [file DataSheet1.ZIP › Supplementary_Materials/result.mstocirc.ath/6draw_circ/ath_circ_008678_circw .png]

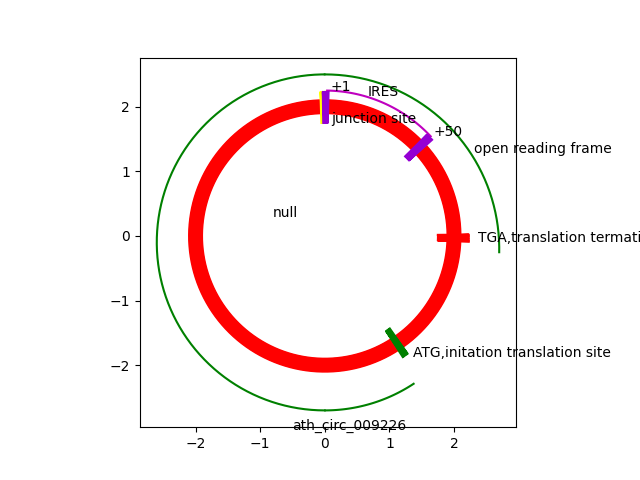

Supplement: Supplementary file 1 [file DataSheet1.ZIP › Supplementary_Materials/result.mstocirc.ath/6draw_circ/ath_circ_009226_circc.png]

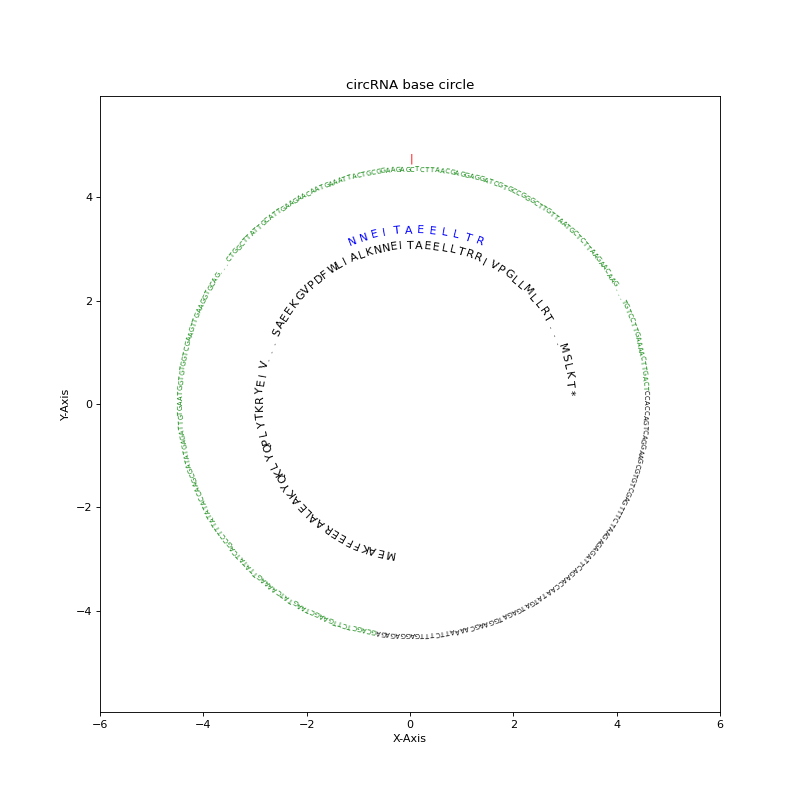

Supplement: Supplementary file 1 [file DataSheet1.ZIP › Supplementary_Materials/result.mstocirc.ath/6draw_circ/ath_circ_009226_circw .png]

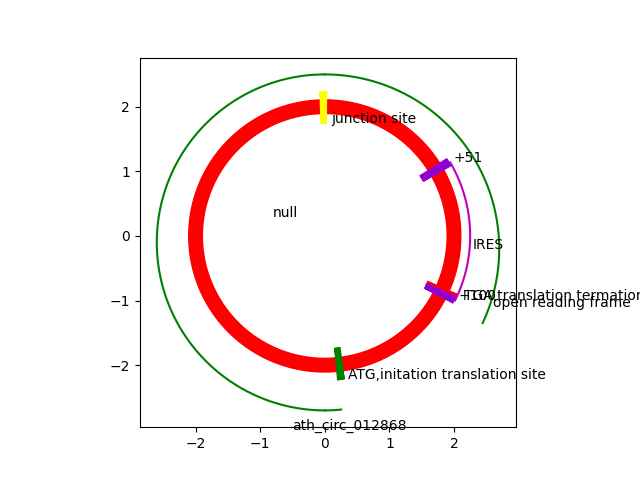

Supplement: Supplementary file 1 [file DataSheet1.ZIP › Supplementary_Materials/result.mstocirc.ath/6draw_circ/ath_circ_012868_circc.png]

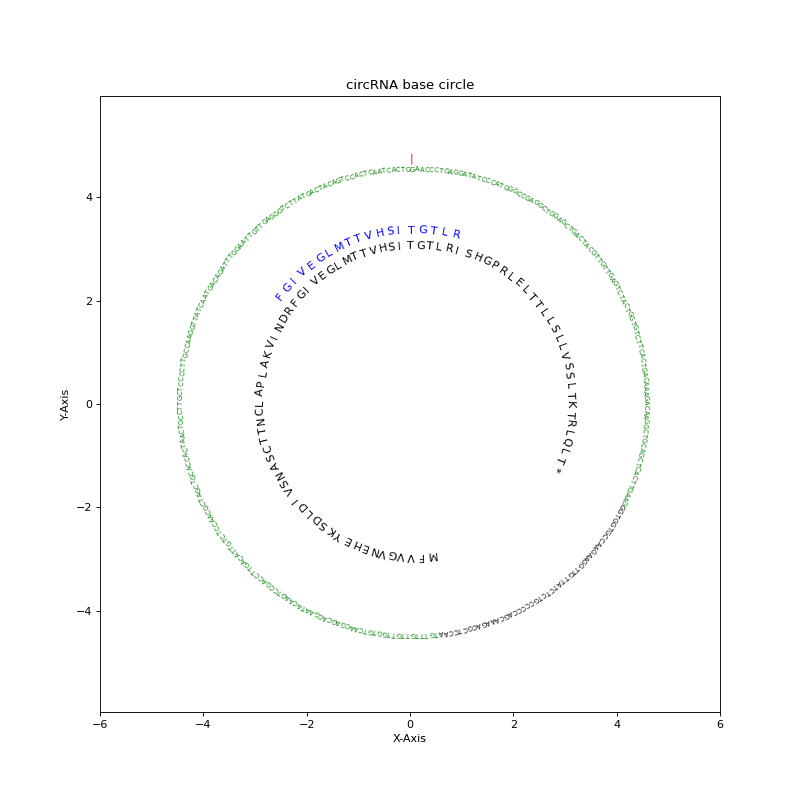

Supplement: Supplementary file 1 [file DataSheet1.ZIP › Supplementary_Materials/result.mstocirc.ath/6draw_circ/ath_circ_012868_circw .png]

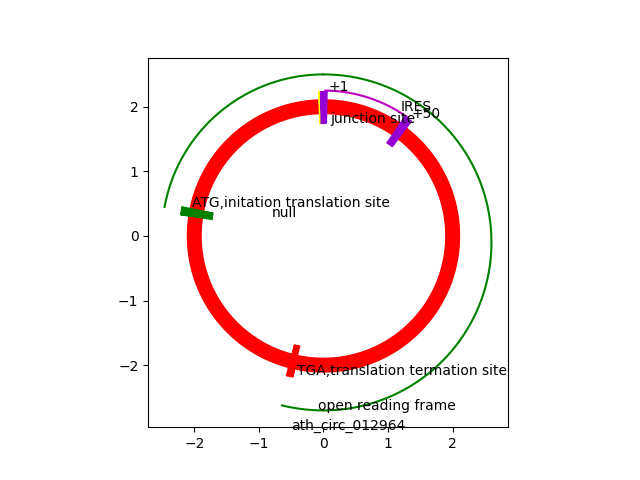

Supplement: Supplementary file 1 [file DataSheet1.ZIP › Supplementary_Materials/result.mstocirc.ath/6draw_circ/ath_circ_012964_circc.png]

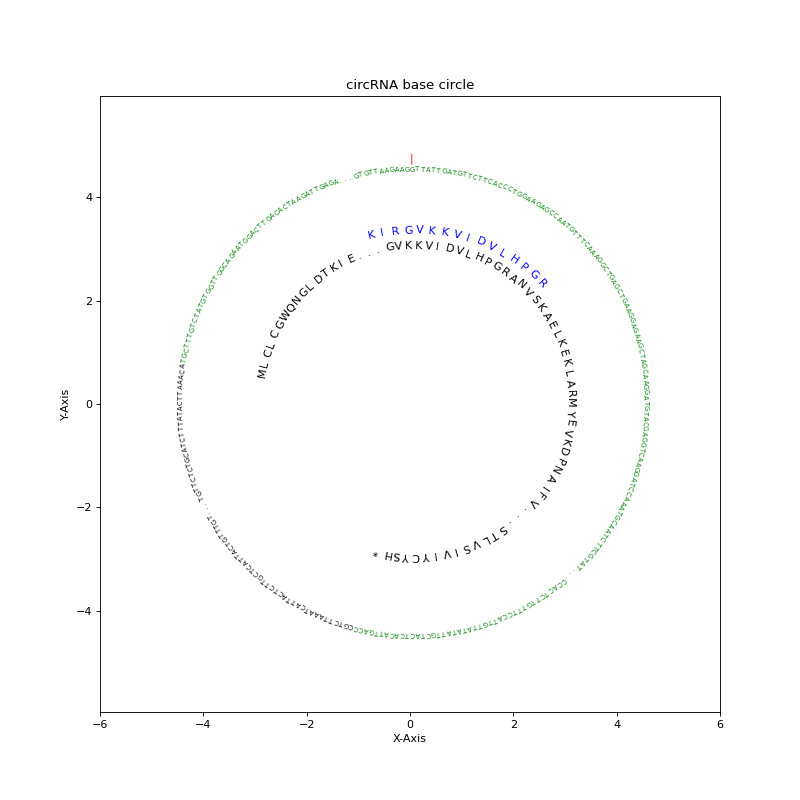

Supplement: Supplementary file 1 [file DataSheet1.ZIP › Supplementary_Materials/result.mstocirc.ath/6draw_circ/ath_circ_012964_circw .png]

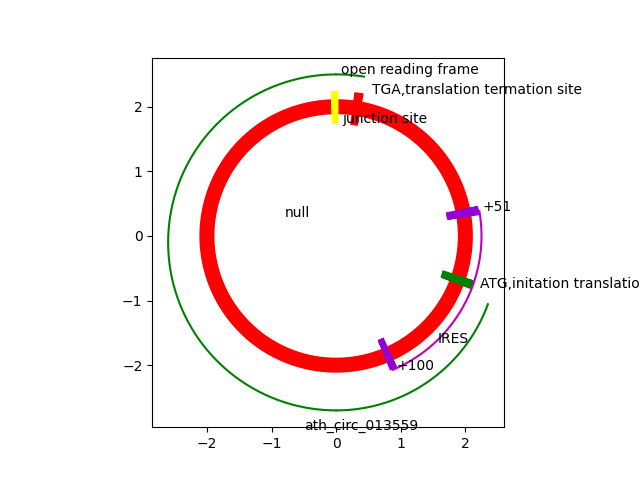

Supplement: Supplementary file 1 [file DataSheet1.ZIP › Supplementary_Materials/result.mstocirc.ath/6draw_circ/ath_circ_013559_circc.png]

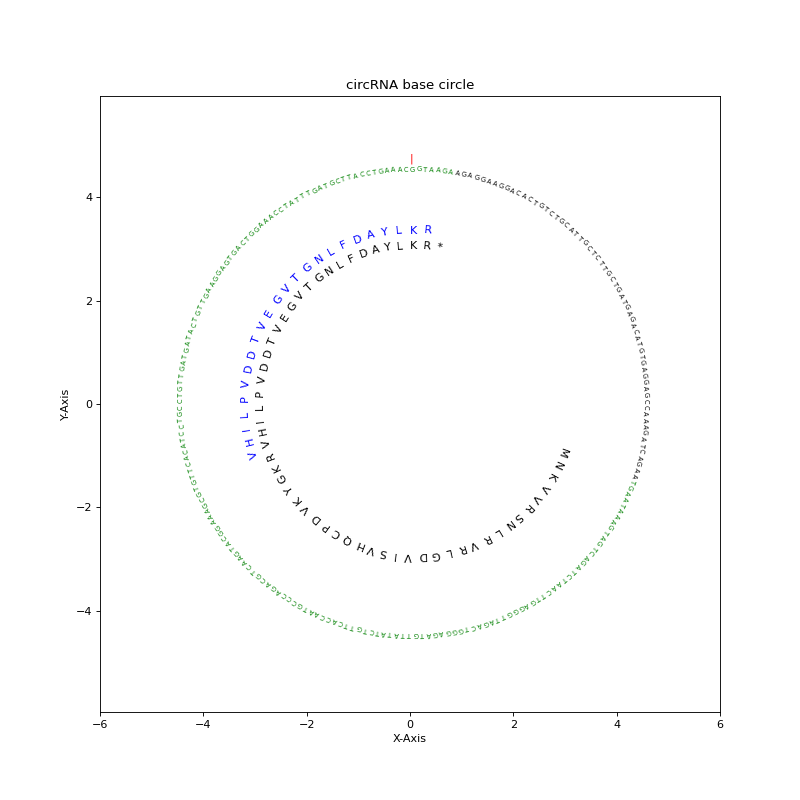

Supplement: Supplementary file 1 [file DataSheet1.ZIP › Supplementary_Materials/result.mstocirc.ath/6draw_circ/ath_circ_013559_circw .png]

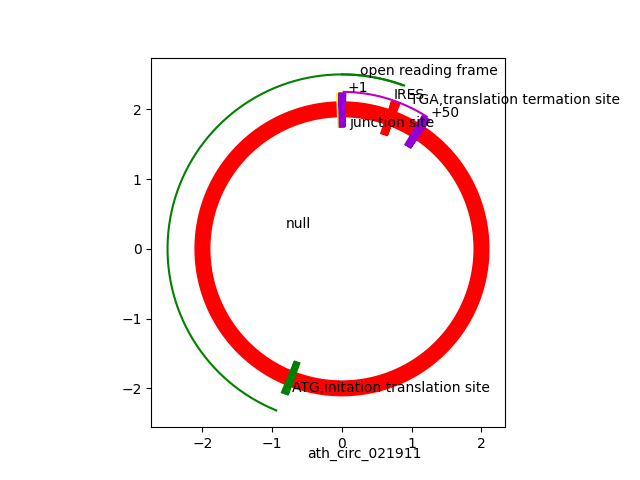

Supplement: Supplementary file 1 [file DataSheet1.ZIP › Supplementary_Materials/result.mstocirc.ath/6draw_circ/ath_circ_021911_circc.png]

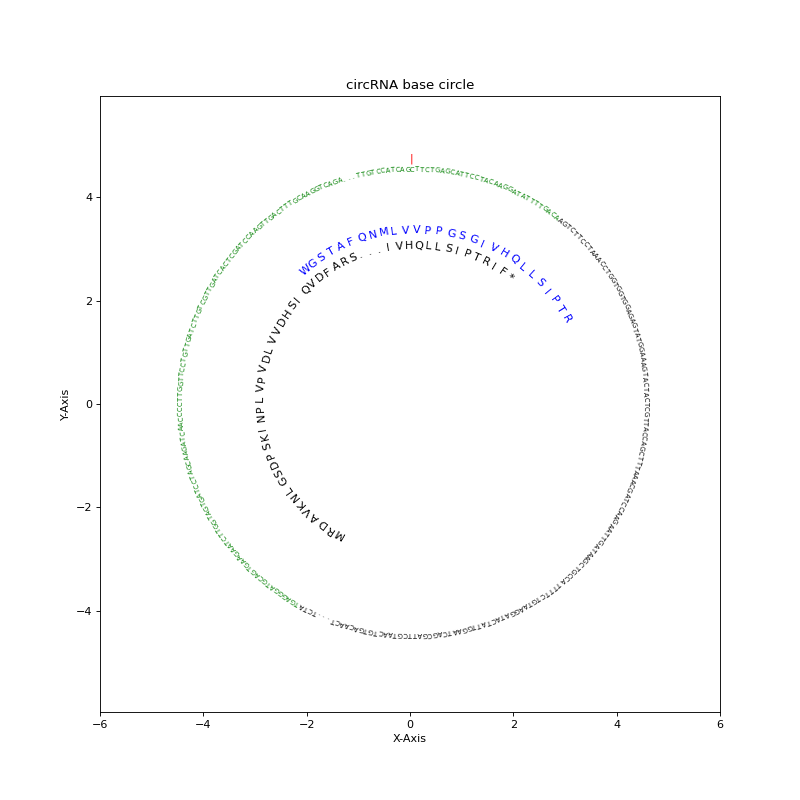

Supplement: Supplementary file 1 [file DataSheet1.ZIP › Supplementary_Materials/result.mstocirc.ath/6draw_circ/ath_circ_021911_circw .png]

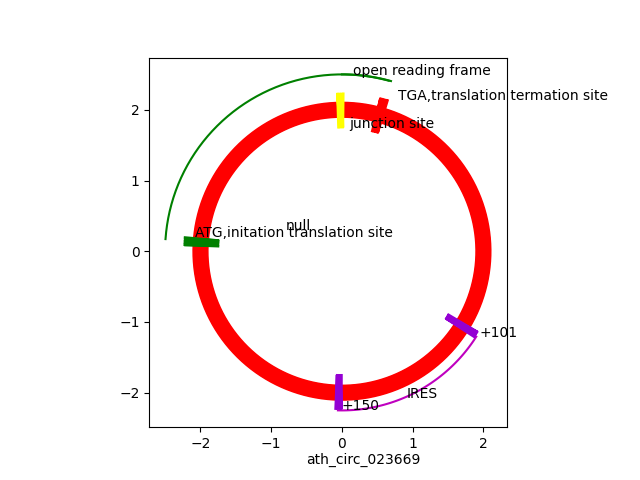

Supplement: Supplementary file 1 [file DataSheet1.ZIP › Supplementary_Materials/result.mstocirc.ath/6draw_circ/ath_circ_023669_circc.png]

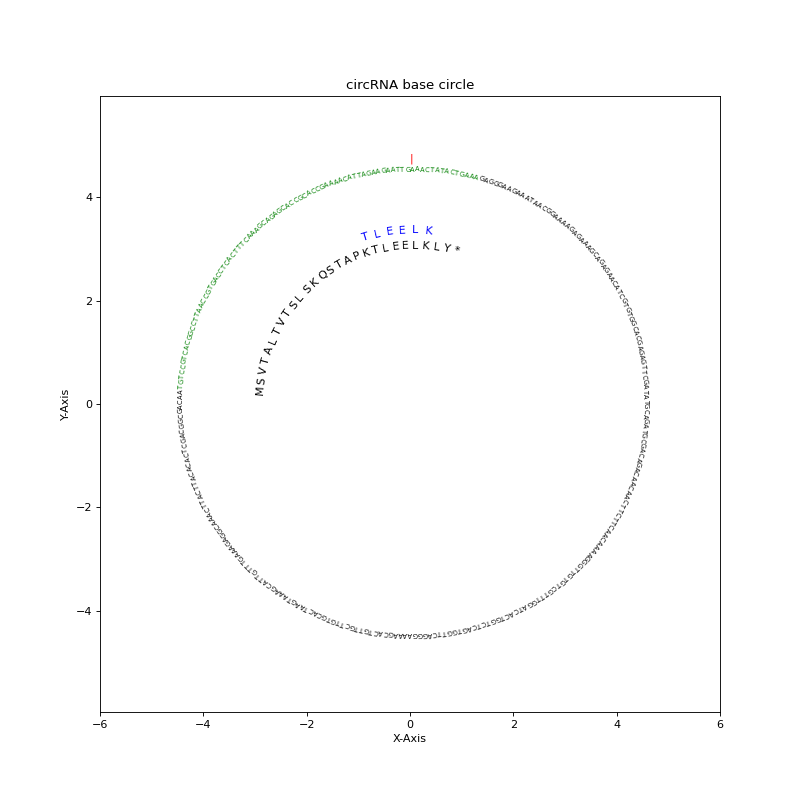

Supplement: Supplementary file 1 [file DataSheet1.ZIP › Supplementary_Materials/result.mstocirc.ath/6draw_circ/ath_circ_023669_circw .png]

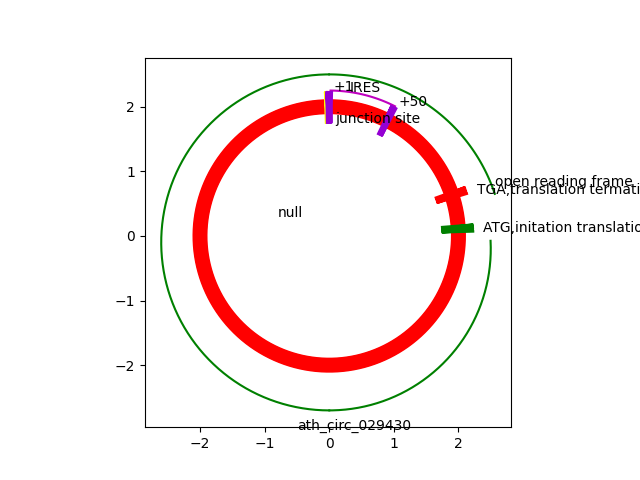

Supplement: Supplementary file 1 [file DataSheet1.ZIP › Supplementary_Materials/result.mstocirc.ath/6draw_circ/ath_circ_029430_circc.png]

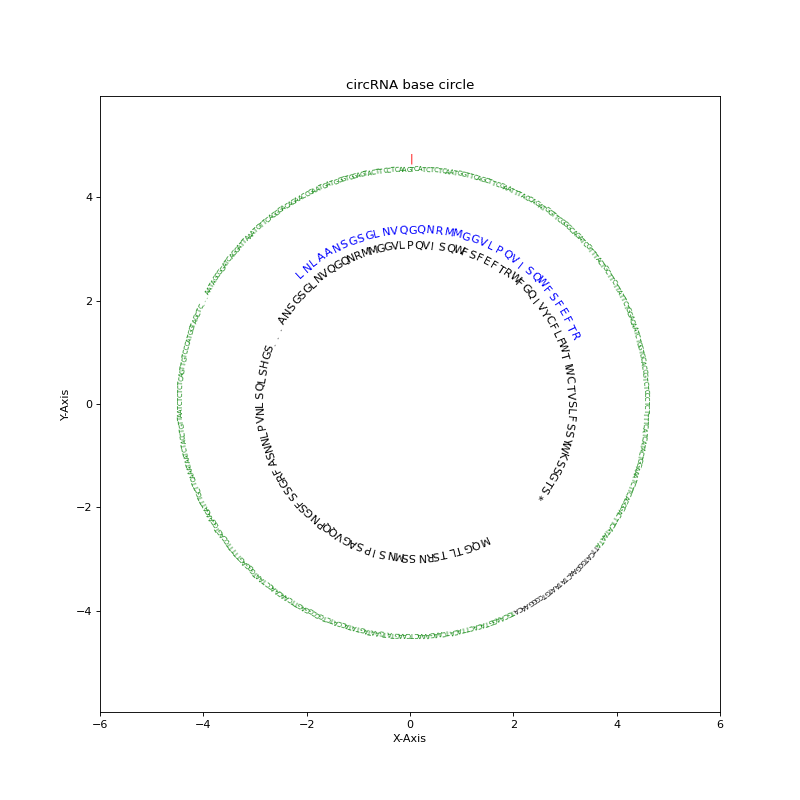

Supplement: Supplementary file 1 [file DataSheet1.ZIP › Supplementary_Materials/result.mstocirc.ath/6draw_circ/ath_circ_029430_circw .png]

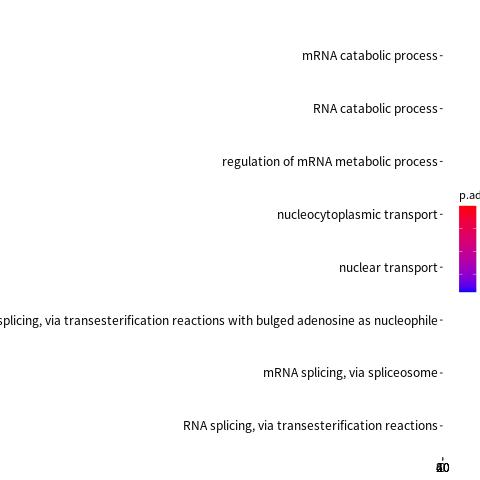

Supplement: Supplementary file 1 [file DataSheet1.ZIP › Supplementary_Materials/result.mstocirc.hsa/5enrich/1.jpg]

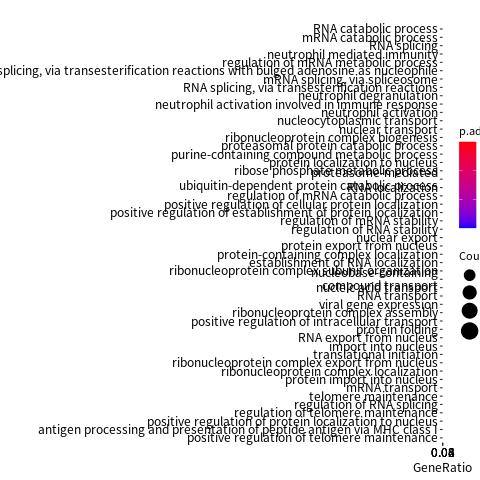

Supplement: Supplementary file 1 [file DataSheet1.ZIP › Supplementary_Materials/result.mstocirc.hsa/5enrich/2.jpg]

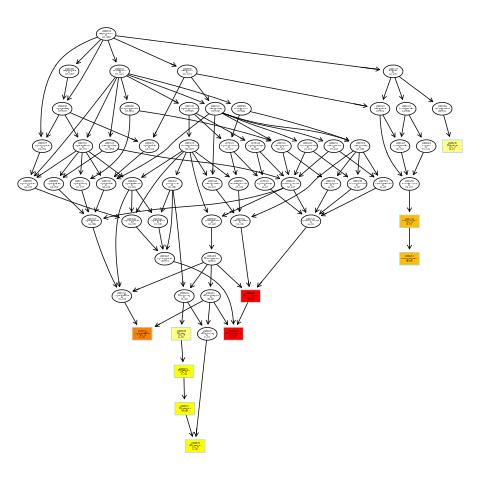

Supplement: Supplementary file 1 [file DataSheet1.ZIP › Supplementary_Materials/result.mstocirc.hsa/5enrich/3.jpg]

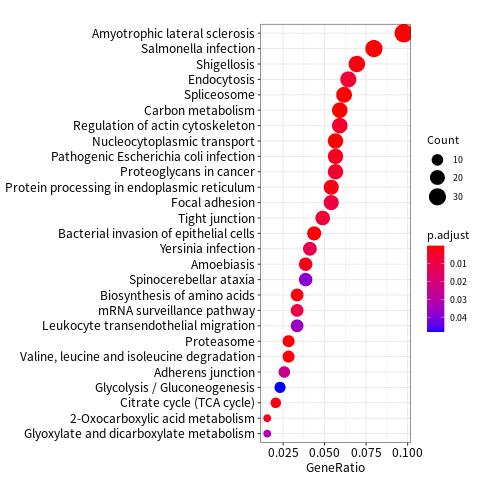

Supplement: Supplementary file 1 [file DataSheet1.ZIP › Supplementary_Materials/result.mstocirc.hsa/5enrich/4.jpg]

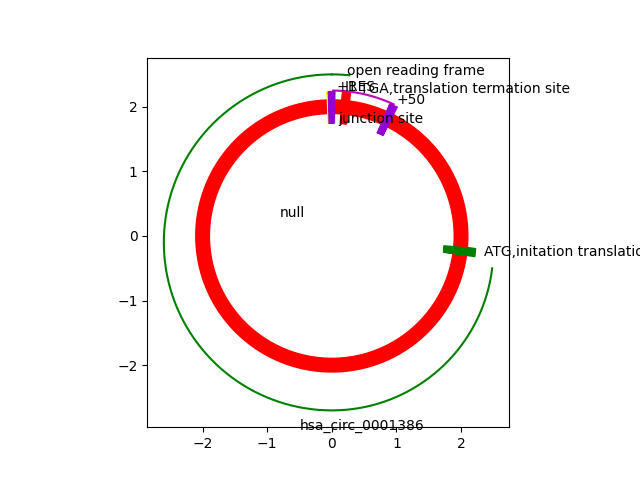

Supplement: Supplementary file 1 [file DataSheet1.ZIP › Supplementary_Materials/result.mstocirc.hsa/6draw_circ/hsa_circ_0001386_circc.png]

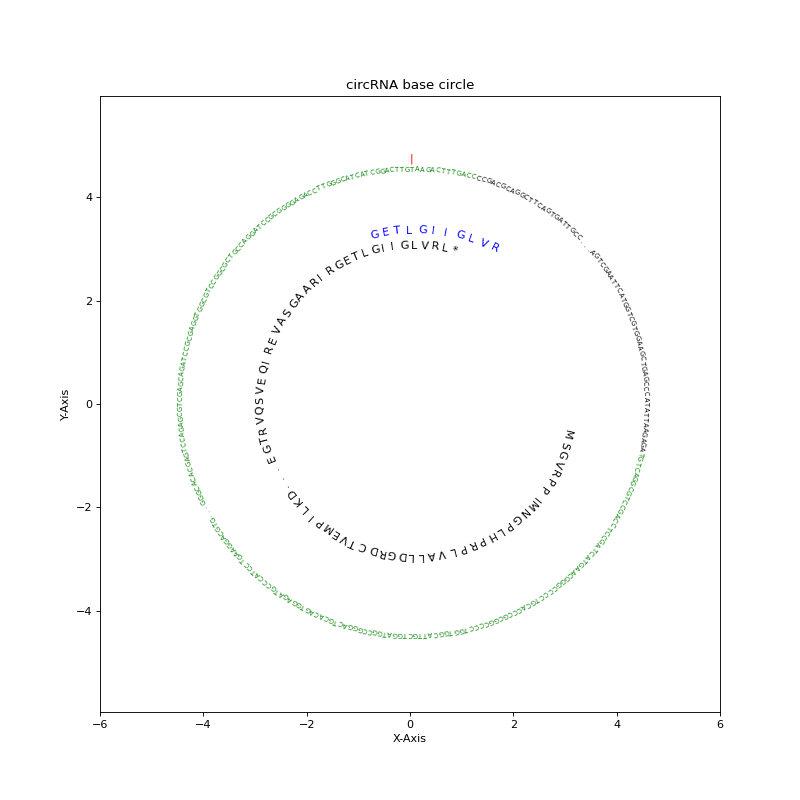

Supplement: Supplementary file 1 [file DataSheet1.ZIP › Supplementary_Materials/result.mstocirc.hsa/6draw_circ/hsa_circ_0001386_circw .png]

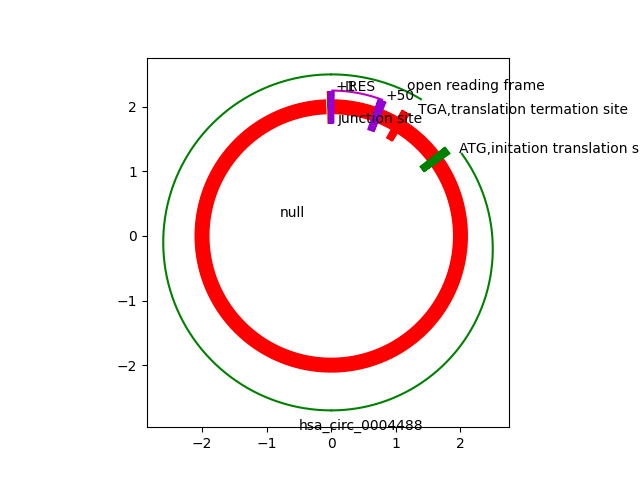

Supplement: Supplementary file 1 [file DataSheet1.ZIP › Supplementary_Materials/result.mstocirc.hsa/6draw_circ/hsa_circ_0004488_circc.png]

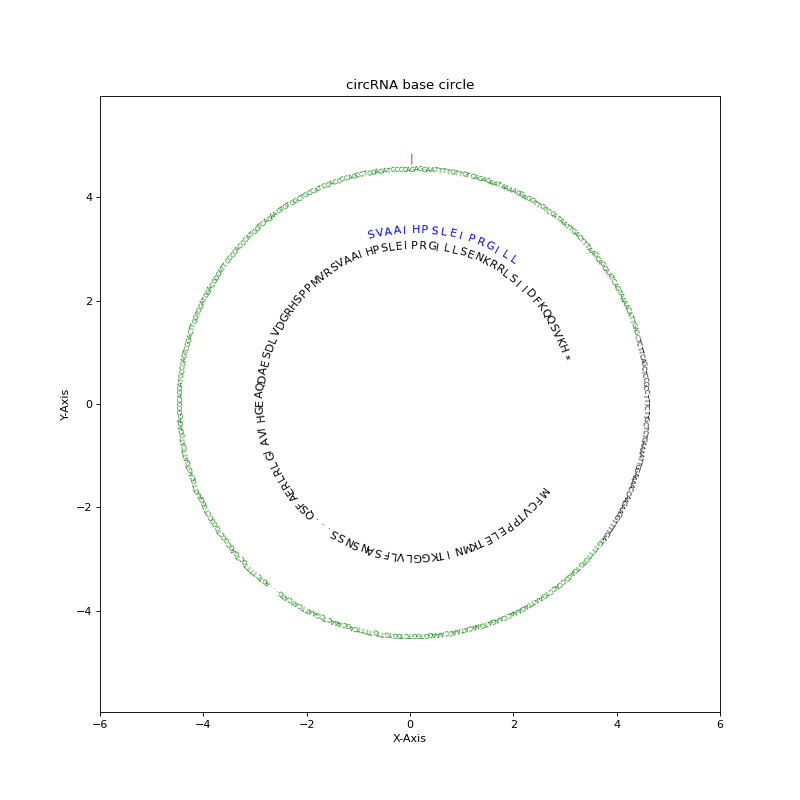

Supplement: Supplementary file 1 [file DataSheet1.ZIP › Supplementary_Materials/result.mstocirc.hsa/6draw_circ/hsa_circ_0004488_circw .png]

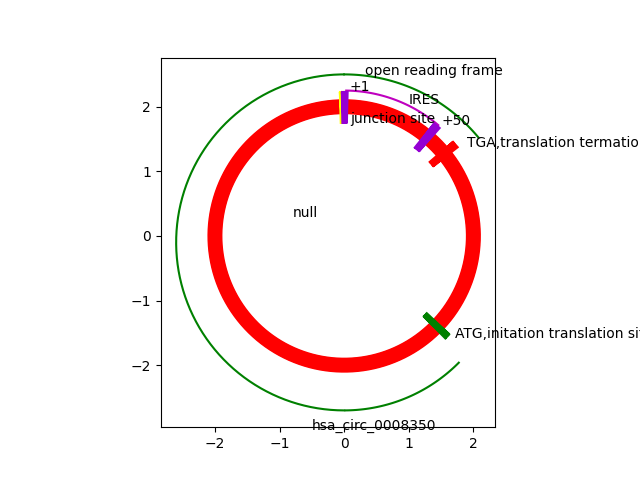

Supplement: Supplementary file 1 [file DataSheet1.ZIP › Supplementary_Materials/result.mstocirc.hsa/6draw_circ/hsa_circ_0008350_circc.png]

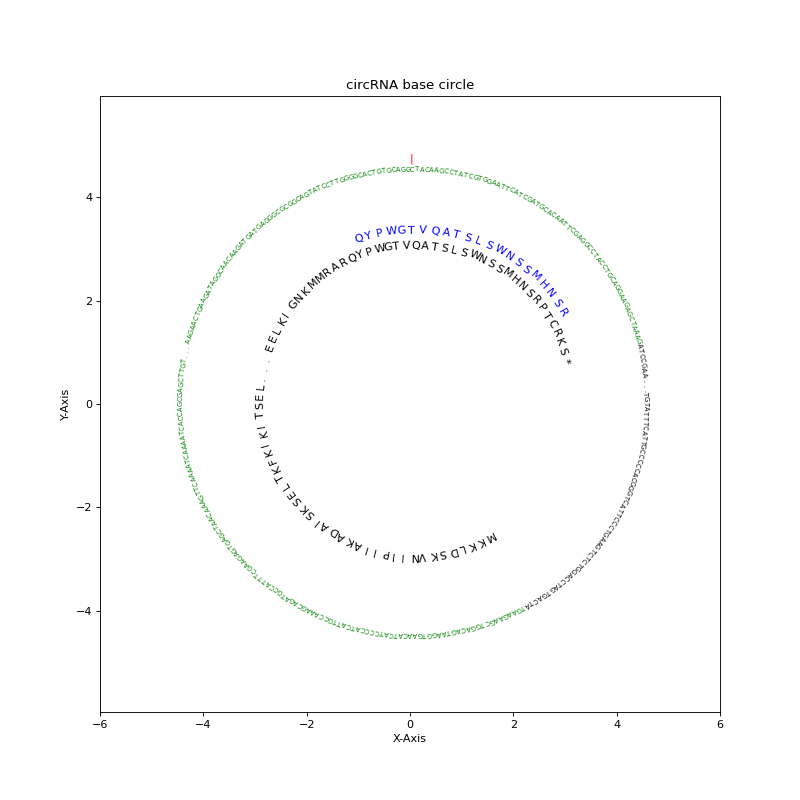

Supplement: Supplementary file 1 [file DataSheet1.ZIP › Supplementary_Materials/result.mstocirc.hsa/6draw_circ/hsa_circ_0008350_circw .png]

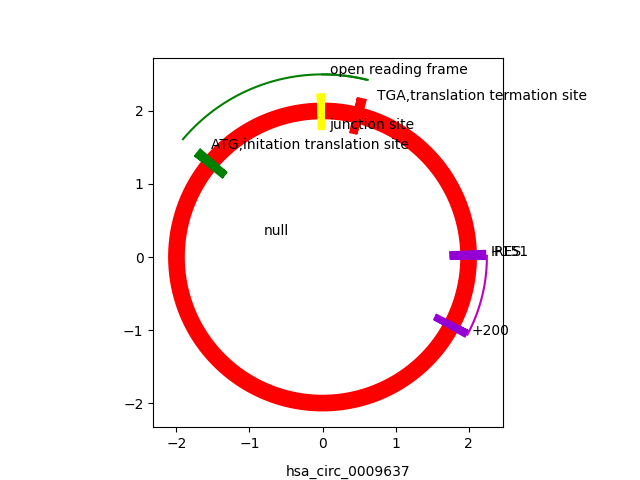

Supplement: Supplementary file 1 [file DataSheet1.ZIP › Supplementary_Materials/result.mstocirc.hsa/6draw_circ/hsa_circ_0009637_circc.png]

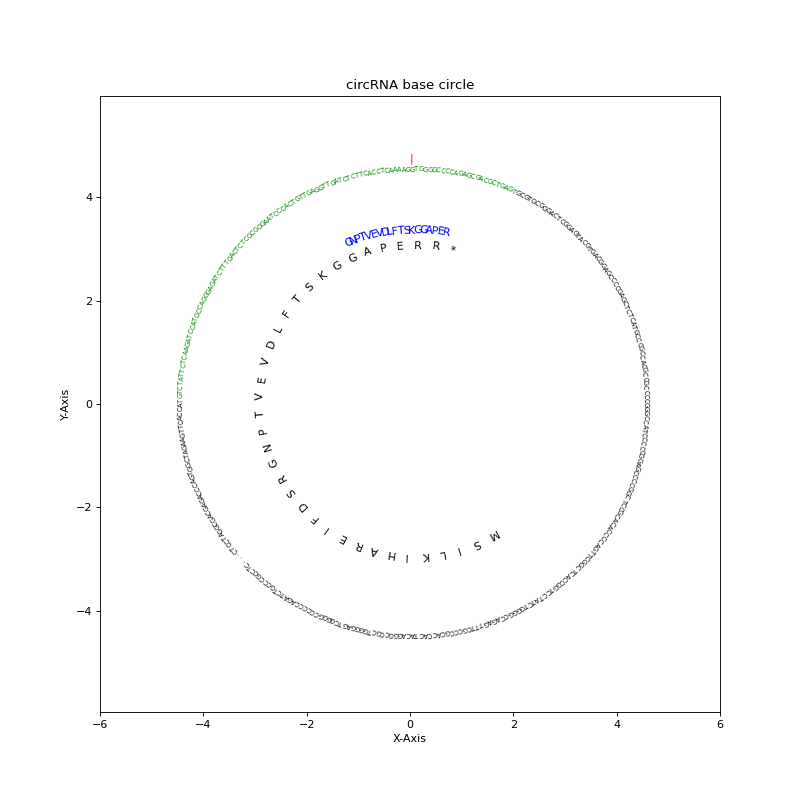

Supplement: Supplementary file 1 [file DataSheet1.ZIP › Supplementary_Materials/result.mstocirc.hsa/6draw_circ/hsa_circ_0009637_circw .png]

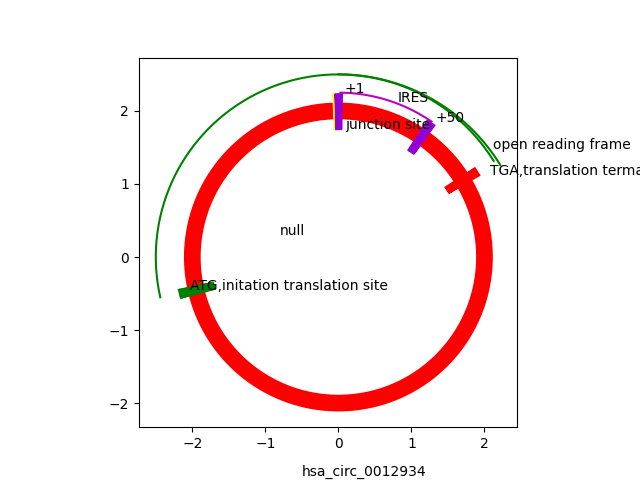

Supplement: Supplementary file 1 [file DataSheet1.ZIP › Supplementary_Materials/result.mstocirc.hsa/6draw_circ/hsa_circ_0012934_circc.png]

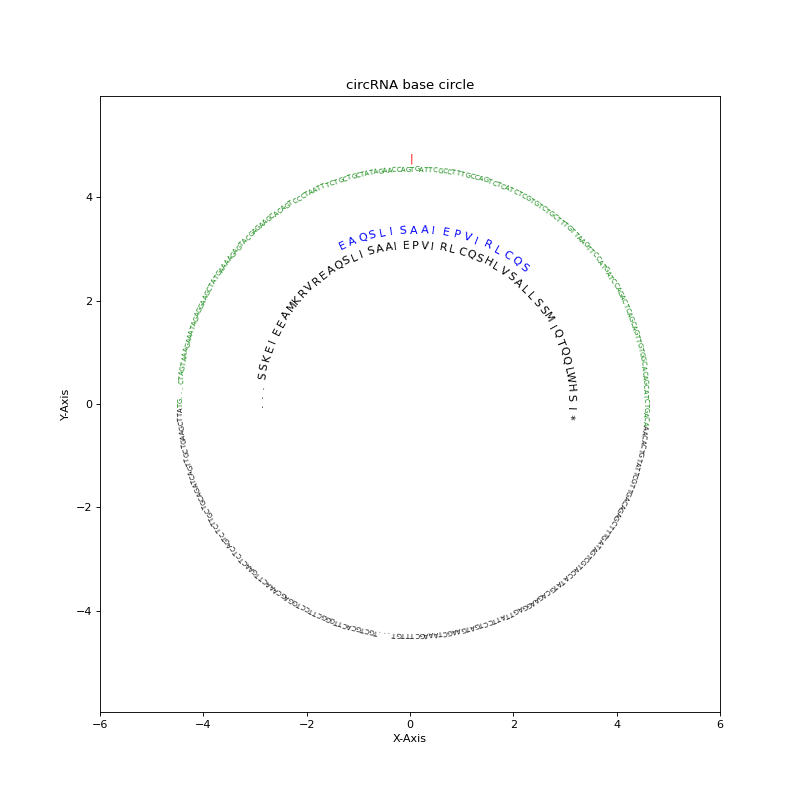

Supplement: Supplementary file 1 [file DataSheet1.ZIP › Supplementary_Materials/result.mstocirc.hsa/6draw_circ/hsa_circ_0012934_circw .png]

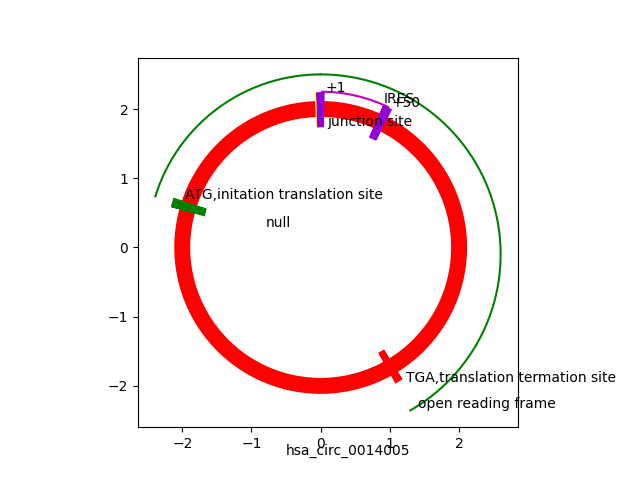

Supplement: Supplementary file 1 [file DataSheet1.ZIP › Supplementary_Materials/result.mstocirc.hsa/6draw_circ/hsa_circ_0014005_circc.png]

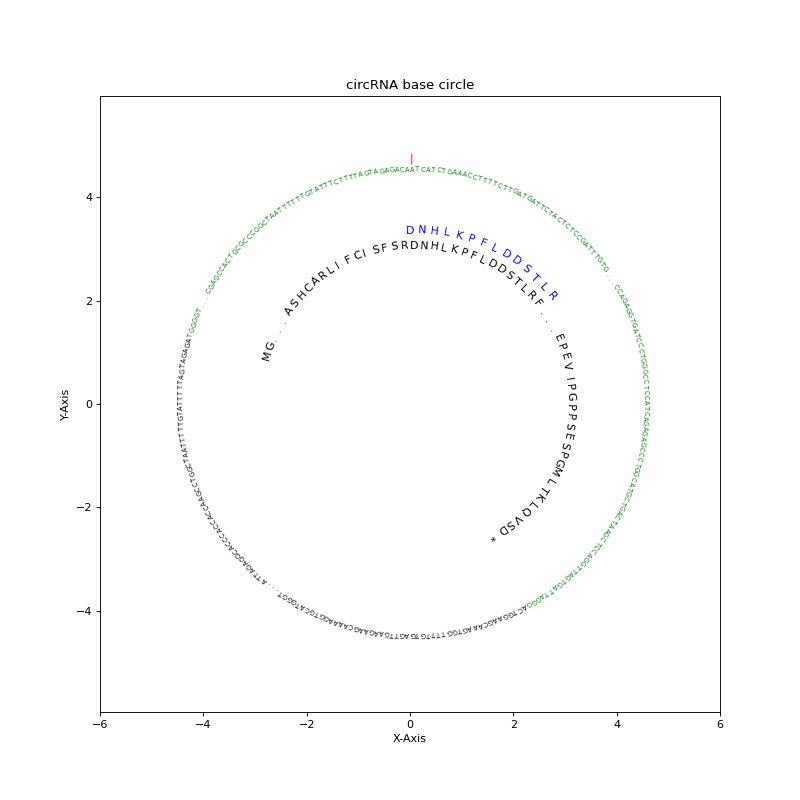

Supplement: Supplementary file 1 [file DataSheet1.ZIP › Supplementary_Materials/result.mstocirc.hsa/6draw_circ/hsa_circ_0014005_circw .png]

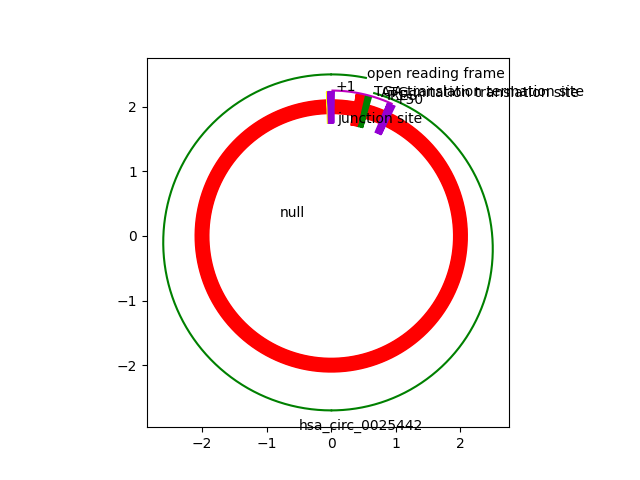

Supplement: Supplementary file 1 [file DataSheet1.ZIP › Supplementary_Materials/result.mstocirc.hsa/6draw_circ/hsa_circ_0025442_circc.png]

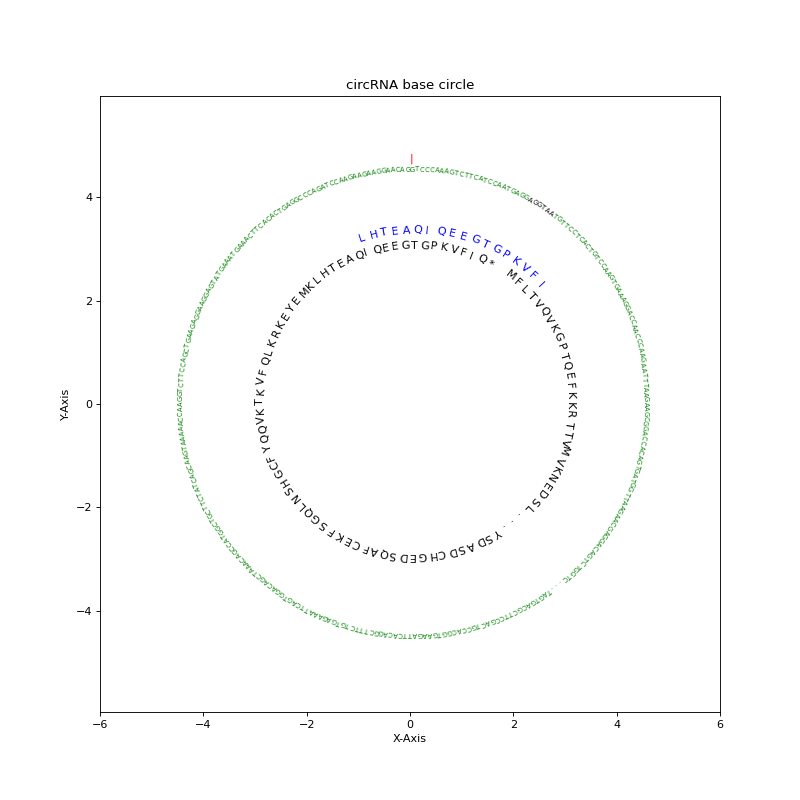

Supplement: Supplementary file 1 [file DataSheet1.ZIP › Supplementary_Materials/result.mstocirc.hsa/6draw_circ/hsa_circ_0025442_circw .png]

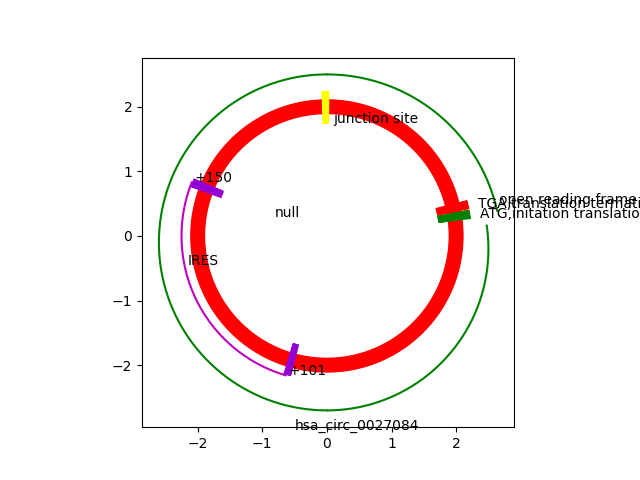

Supplement: Supplementary file 1 [file DataSheet1.ZIP › Supplementary_Materials/result.mstocirc.hsa/6draw_circ/hsa_circ_0027084_circc.png]

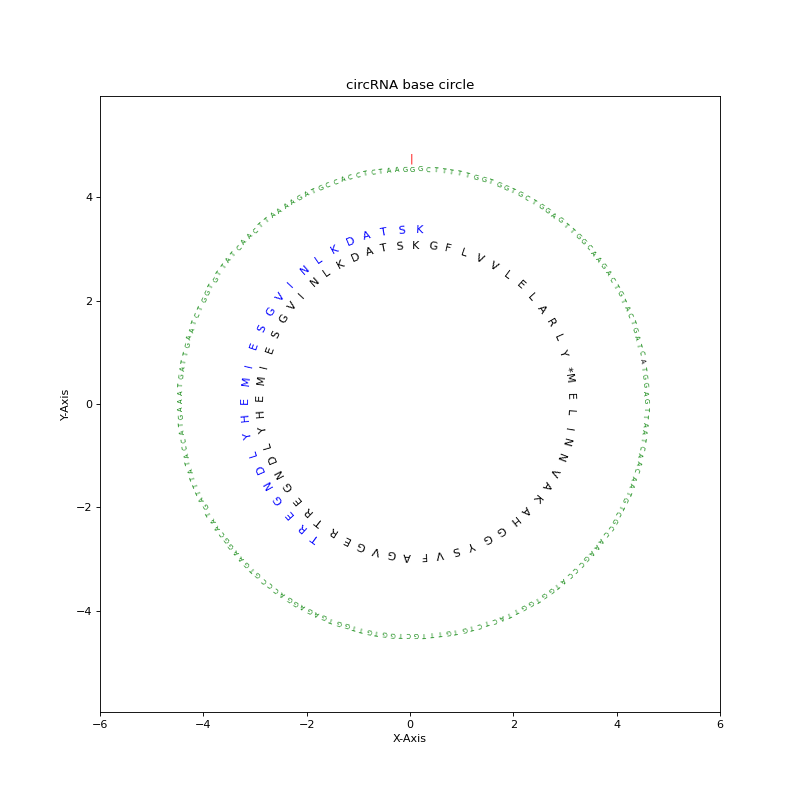

Supplement: Supplementary file 1 [file DataSheet1.ZIP › Supplementary_Materials/result.mstocirc.hsa/6draw_circ/hsa_circ_0027084_circw .png]

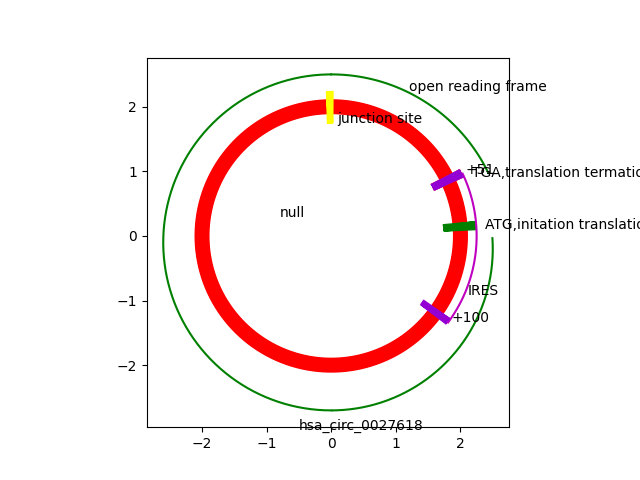

Supplement: Supplementary file 1 [file DataSheet1.ZIP › Supplementary_Materials/result.mstocirc.hsa/6draw_circ/hsa_circ_0027618_circc.png]

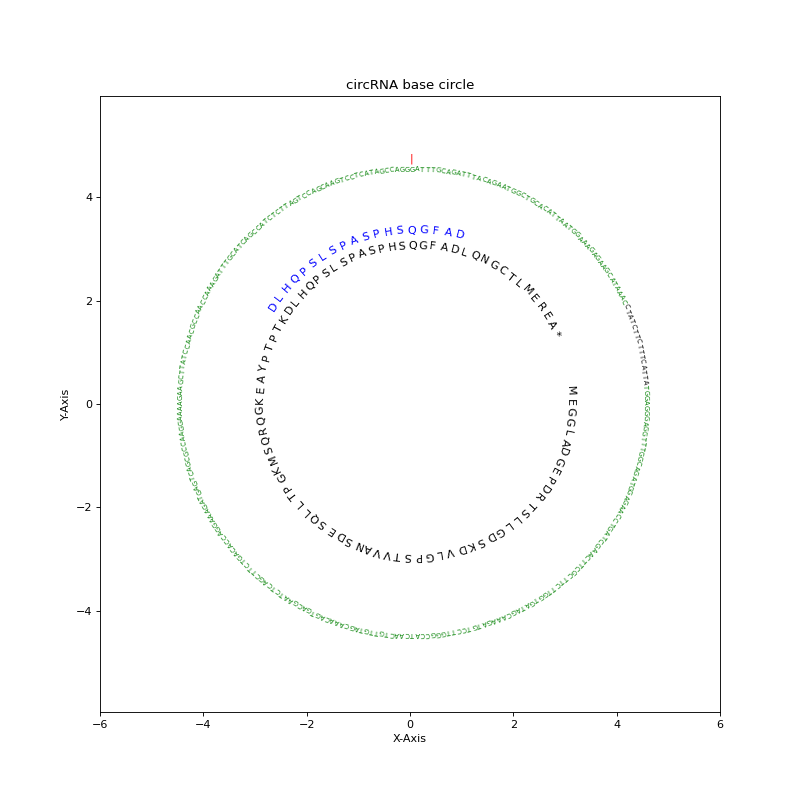

Supplement: Supplementary file 1 [file DataSheet1.ZIP › Supplementary_Materials/result.mstocirc.hsa/6draw_circ/hsa_circ_0027618_circw .png]

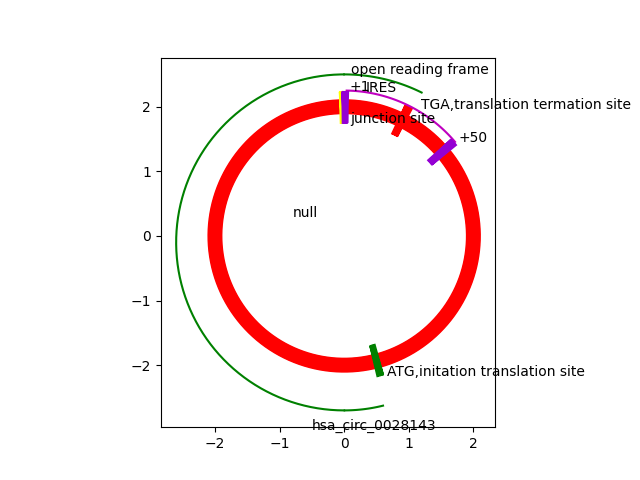

Supplement: Supplementary file 1 [file DataSheet1.ZIP › Supplementary_Materials/result.mstocirc.hsa/6draw_circ/hsa_circ_0028143_circc.png]

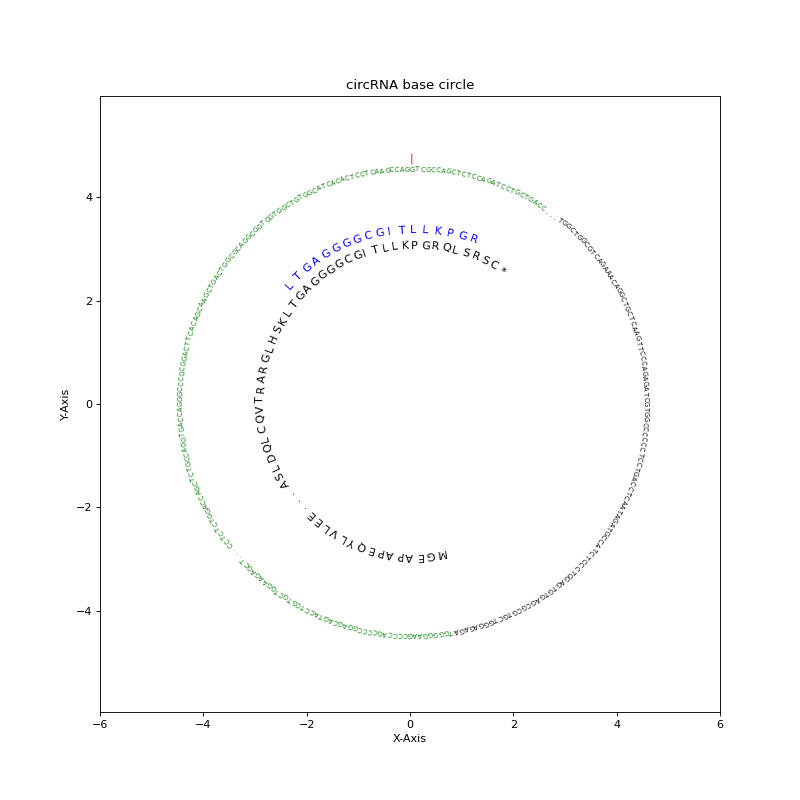

Supplement: Supplementary file 1 [file DataSheet1.ZIP › Supplementary_Materials/result.mstocirc.hsa/6draw_circ/hsa_circ_0028143_circw .png]
